# YIPF2 is a novel Rab-GDF that enhances HCC malignant phenotypes by facilitating CD147 endocytic recycle

**DOI:** 10.1038/s41419-019-1709-8

**Published:** 2019-06-12

**Authors:** Shanshan Qi, Linjia Su, Jing Li, Pu Zhao, Qing Zhang, Xiuran Niu, Jingyuan Liu, Guhe Jia, Xiaoxuan Wei, Jan Tavernier, Jianli Jiang, Zhinan Chen, Sihe Zhang

**Affiliations:** 1 Department of Cell Biology, School of Medicine, Nankai University, Tianjin, China; 2 Department of Neurobiology, College of Life and Health Sciences, Northeastern University, Shenyang, China; 3 Department of Clinical Laboratory, Cancer Hospital of Tianjin Medical University, Tianjin, China; 4 Cytokine Receptor Laboratory, VIB Medical Biotechnology Center, Ghent University, Ghent, Belgium; 5 Department of Cell Biology, National Translational Science Center for Molecular Medicine, State Key Laboratory of Cancer Biology, Fourth Military Medical University, Xi’an, China

**Keywords:** Exocytosis, Liver cancer

## Abstract

An increased surface level of CIE (clathrin-independent endocytosis) proteins is a new feature of malignant neoplasms. CD147 is a CIE glycoprotein highly up-regulated in hepatocellular carcinoma (HCC). The ability to sort out the early endosome and directly target the recycling pathway confers on CD147 a prolonged surface half-life. However, current knowledge on CD147 trafficking to and from the cell-surface is limited. In this study, an MSP (membrane and secreted protein)-cDNA library was screened against EpoR/LR-F3/CD147EP-expressed cells by MAPPIT (mammalian protein–protein interaction trap). CD147 co-expressing with the new binder was investigated by GEPIA (gene expression profiling interactive analysis). The endocytosis, ER-Golgi trafficking and recycling of CD147 were measured by confocal imaging, flow cytometry, and biotin-labeled chase assays, respectively. Rab GTPase activation was checked by GST-RBD pull-down and MMP activity was measured by gelatin zymography. HCC malignant phenotypes were determined by cell adhesion, proliferation, migration, Transwell motility, and invasion assays. An ER-Golgi-resident transmembrane protein YIPF2 was identified as an intracellular binder to CD147. YIPF2 correlated and co-expressed with CD147, which is a survival predictor for HCC patients. YIPF2 is critical for CD147 glycosylation and trafficking functions in HCC cells. YIPF2 acts as a Rab-GDF (GDI-displacement factor) regulating three independent trafficking steps. First, YIPF2 recruits and activates Rab5 and Rab22a GTPases to the endomembrane structures. Second, YIPF2 modulates the endocytic recycling of CD147 through distinctive regulation on Rab5 and Rab22a. Third, YIPF2 mediates the mature processing of CD147 via the ER-Golgi trafficking route. Decreased YIPF2 expression induced a CD147 efficient delivery to the cell-surface, promoted MMP secretion, and enhanced the adhesion, motility, migration, and invasion behaviors of HCC cells. Thus, YIPF2 is a new trafficking determinant essential for CD147 glycosylation and transport. Our findings revealed a novel YIPF2-controlled ER-Golgi trafficking signature that promotes CD147-medated malignant phenotypes in HCC.

## Introduction

The aberrant expression and distribution of membrane antigens can create diverse malignant phenotypes in neoplasms^1,2^. Membrane antigens are processed in ER-Golgi biosynthetic network. Subsequently, they are delivered to cell-surface. ligand or antibody binding can activate or cluster membrane antigens, and stimulate their uptake through clathrin-mediated endocytosis (CME) or clathrin-independent endocytosis (CIE)^3^. Internalized antigens explore a series of sorting events. Then, they are either recycled back to cell-surface for another round of trafficking, or sorted to lysosomes or to proteasome for degradation, leading to irreversible termination of antigen-mediated functions^4^.

CD147 antigen is a type I transmembrane glycoprotein that belongs to immunoglobulin superfamily. Mature CD147 is an N-linked glycosylated protein and exists both in transmembrane and soluble forms in tumors^5–8^. Depending on the degree of glycosylation, CD147 is Western-blotted in two smear bands (32–65 kDa), suggesting it exists in two forms: high-glycosylated CD147 (HG-CD147) and low-glycosylated CD147 (LG-CD147). Hepatocellular carcinoma (HCC) is one of the most lethal and prevalent cancers worldwide. We previously showed that CD147 is up-regulated in HCC, which promotes hepatocarcinogenesis, apoptosis inhibition, invasion, and distant metastasis^9,10^.

A complex regulation network controls CD147 synthesis and transport^5–8^. The endocytosis and recycling of CD147 have critical roles in tumor progression^11^. Most studies revealed that CD147 is internalized through Rab5-activation-dependent CIE, which is associated with tubular endosomes and by-passes the merging with EEA1-positive endosomes^12–15^. Activation of Rab22a or Arf6 GTPases can further promote CD147trafficking, accelerating it entering the fast recycling pathway^12,16,17^. This might avoid the slow recycling pathway through Rab11-positive endosomes or the degradation pathway for CD147^15,18^. It is evident that CD147 binds with different partners during its transport in cancer cells. For example, caveolin-1 associates with LG-CD147, restrict its biosynthetic conversion to HG-CD147, and escorts LG-CD147 to cell surface^19,20^. Monocarboxylate transporters tightly associate with CD147 and influence its processing in Golgi as well as trans-localization to cell surface^21,22^. Cyp60 binding with CD147, assists CD147 transport from Golgi lumen to cell surface^23,24^. Other proteins, such as Gal-3 and Annexin A2, can also associate with CD147, affecting its clustering or trafficking in vesicles^25–27^. Although these proteins are involved in subcellular transport of CD147, none were found to be key regulator in its endocytic recycling process. In addition, previous identification of CD147 binders was performed in cell lysates or screened by yeast two-hybrid. That most likely missed the natural binders to CD147 during its trafficking.

In this study, we screened intracellular binders of CD147 using mammalian protein–protein interaction trap (MAPPIT)^28^. Interestingly, one of CD147 binders, YIPF2, was found to be a Rab-GDF recruiting and activating Rab5/Rab22a on ER-Golgi, promoting endocytosis, recycling, and secretion of CD147, and thus enhancing the malignant phenotypes of HCC cells. This benefits our understanding of the regulation mechanism on CD147 trafficking, and also supplies a target to disrupt the CD147 transport pathway in HCC progression.

## Results

### YIPF2 is a new intracellular binder to CD147

**Fig. 1:**
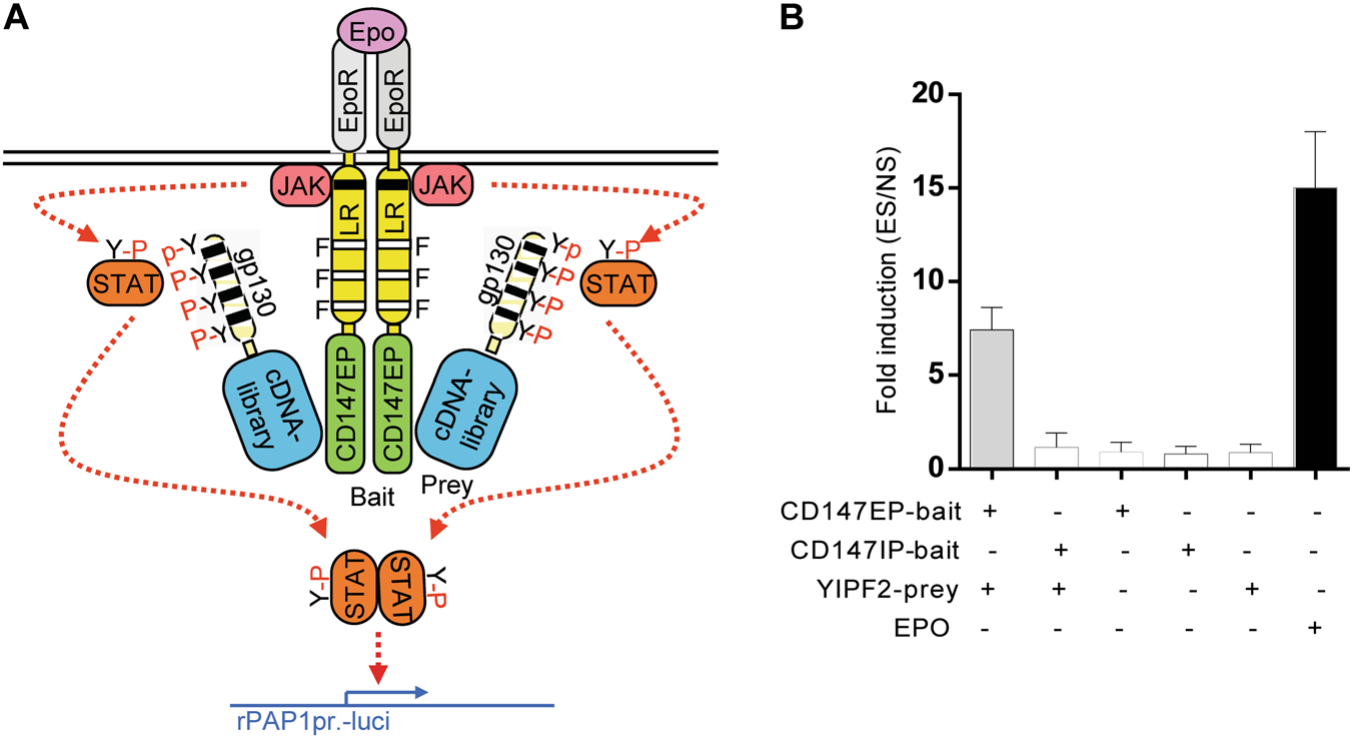
YIPF2 is an intracellular binder to CD147 screened by MAPPIT. **a** Principle of screening MAPPIT. A heterologous bait CD147EP was fused to a chimera receptor consisting of the extracellular domain of homo-dimeric EpoR fused to the transmembrane and cytosolic domains of the LR-F3. Tyrosine to phenylalanine mutations (F3) eliminated the functional STAT3 recruitment sites at positions 1138, 985, and 1077 linking to the Ras pathway and to JAK-STAT signaling inhibitors. Heterologous prey polypeptides, i.e., MSP-cDNA library, were fused to the receptor fragment of gp130 containing four functional STAT3 recruitment sites. If bait and prey bound, the recruited receptor fragment was phosphorylated upon ligand-induced receptor activation, and a STAT3-dependent reporter gene signal was obtained. **b** Determining the binding of YIPF2 with CD147EP/IP by analytical MAPPIT. HEK293T cells were co-transfected with the pSEL1/CD147EP or pSEL1/CD147IP (bait) plasmid together with the pMG1/YIPF2 (prey) plasmid or a mock plasmid, and combined with the pXP2d2-rPAP1-luci plasmid. The transfected cells were stimulated for 24 h with EPO (ES) or were not stimulated (NS). Luciferase measurements were performed in triplicate, and positive interactions were considered significant if ES/NS > 5

As CD147-stimulated MMP (matrix metalloproteinase) secretion involves its interaction with membrane and secreted protein (MSP) on neighboring cells^5,7,8^, we first established a human liver cancer-derived MSP-cDNA library (Supplementary Fig. 1). To screen the trafficking binders of CD147 under physiological condition, HEK293-16 cells expressing EpoR/LR-F3/CD147EF chimera receptor (as bait) were infected with retroviral MSP-cDNA library (as prey) (Fig. 1a). After double selection with puromycin and Epo, eighteen clones that survived were screened. They contained six unique gene candidates (Supplementary Fig. 2a, b). Checking their function in UniProt database found that the yeast homolog of YIPF2 (Yif1p) is a Rab-bound protein involving vesicle transport (Table 1).

**Table 1:** MAPPIT-screened binder to CD147

Clones	Protein	Molecular mass (kd)	Description	Molecular function (from UniProt)
1	YIPF2	35	Yip1 domain family,member 2	Rab GTPase binding
2	SCAP	140	Sterol regulatory element-binding protein cleavage-activating protein	Escort protein required for cholesterol as well as lipid homeostasis. Regulates export of the SCAP/SREBF complex from the ER upon low cholesterol
3	MTG1	37	Mitochondrial ribosome-associated GTPase 1	Regulates the mitochondrial ribosome assembly and of translational activity. Displays GTPase activity
4	CISH	29	Cytokine-inducible SH2-containing protein	Involved in the negative regulation of cytokines that signal through the JAK-STAT5 pathway; protein kinase inhibitor activity
5	PPCS	34	Phosphopantothenoylcysteine synthetase	Catalyzes the first step in the biosynthesis of coenzyme A from vitamin B5. phosphopantothenate–cysteine ligase activity
6	NARF	51	Nuclear prelamin A recognition factor	Lamin binding; NADH dehydrogenase activity

**Fig. 2:**
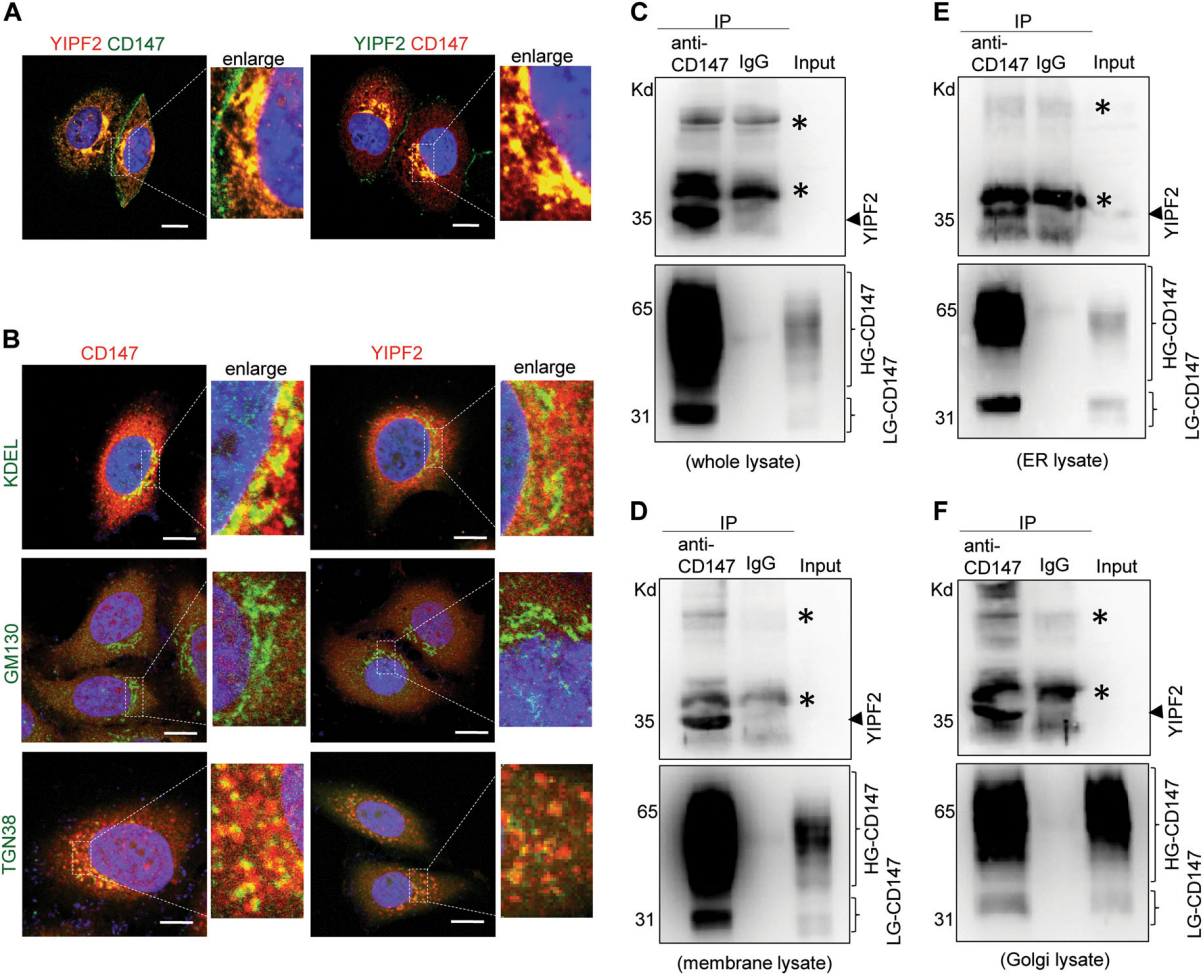
YIPF2 co-localizes with CD147 at ER-Golgi organelles in HCC cells. **a** HepG2 cells were PFA-fixated, samponi-permeabilized, and stained with Ab combinations. Left: anti-YIPF2 Ab plus anti-rabbit Ab-AF594 (red color), H18Ab plus anti-mouse Ab-AF488 (green color). Right: anti-YIPF2 Ab plus anti-mouse Ab-AF488 (green color), anti-CD147 pcAb plus anti-rabbit Ab-AF594 (red color). **b** Confocal imaging of the organelle localization of YIPF2 and CD147. HepG2 cells were stained with Ab combinations: anti-YIPF2 Ab (red color, right panel) or anti-CD147 pcAb (red color, left panel) together with anti-KDEL Ab, anti-GM130 Ab and TGN38 Ab, respectively (both with anti-mouse Ab-AF488, green color). Representative observations are shown in (**a**–**b**). Scale bar: 20 μm. **c**–**f** co-IP analysis of the binding of YIPF2 with CD147. CD147 was immunoprecipitated from subcellular fractions (**c** whole lysate. **d** membrane lysate. **e** ER lysate. **f** Golgi lysate.) of HepG2 cells using H18 Ab, and co-immunoprecipitated YIPF2 were blotted with its Ab

To further identify their interaction, extracellular and intracellular portions of CD147 (CD147EP and CD147IP) were included in analytical MAPPIT (Supplementary Fig. 2c), and specific binding of YIPF2 to CD147EP were detected (Fig. 1b). Confocal imaging of their distribution in HepG2 cells demonstrated that YIPF2 was co-localized with CD147 mainly at ER-Golgi network, sorting, and recycling endosomes (Fig. 2a, Supplementary Fig. 4). Furthermore, the co-localization between endogenous CD147/YIPF2 and ER marker KDEL, and Golgi markers GM130 (marking cis-Golgi) and TGN38 (marking trans-Golgi) were determined. Strong co-localized YIPF2-CD147 was observed in ER and trans-Golgi network, whereas their co-localizations were not found in cis-Golgi, plasma membrane, or other membranous organelles (Figs 2b, 4a, Supplementary Figs. 4, 6, 7, 8b, and 9). In addition, co-IP results also validated the specific binding of YIPF2 with CD147 in cell lysates and membranous organelles (Fig. 2c–f). These results indicated that YIPF2 is a new binder to CD147, which is possibly involved in intracellular transport of CD147 via ER-Golgi biosynthetic network.

### Clinical significance of YIPF2-CD147 co-expressed in HCC

**Fig. 3:**
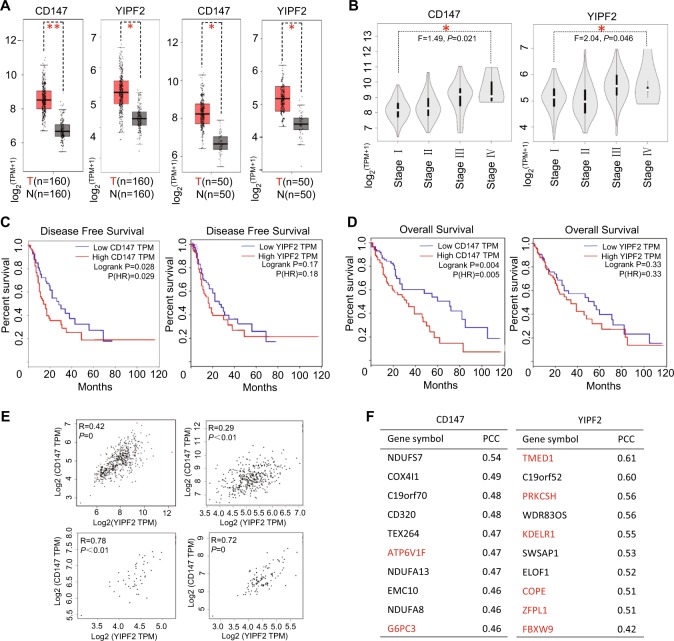
Clinical significance of CD147 and YIPF2 co-expression in HCC. **a** Box plots depict the expression level difference between HCC samples (T) and normal tissues (N). Matched TCGA normal & GTEx data (left two plots) and matched TCGA normal data (right two plots) are respectively shown. Each dot represents expression of a sample. Student’s *t*-test, *p < 0.05, **p< 0.01. **b** Stage plots depict the expression level variation among different pathological stages of HCC tissue samples. **c** Survival plots depict the disease-free survival (DFS) for low and high expression groups of the CD147 gene (left plots) and the YIPF2 gene (right plots) among HCC patients (cutoff: median). **d** Survival plots depict the overall survival (OS) for lo and, high expression groups of the CD147 gene (left plots) and the YIPF2 gene (right plots) among HCC patients (cutoff: median). **e** Pair-wise gene expression correlation analysis for the given sets of TCGA and/or GTEx expression data (left-upper: all mixed, right-upper: HCC tumor, left-below: HCC-paired normal tissues, right-below: liver). Pearson’s *R* test. **f** Co-expression network analysis of function-related genes was based on the datasets across all tumor samples and paired normal tissues. The lists show the top ten genes with functional connections

To investigate the clinical significance of YIPF2-CD147 interaction, we next analyzed their expression by bioinformatics. Although CD147 and YIPF2 tend to be high-expressed in liver cancer patients, CD147 exhibited a higher transcription level in HCC tissues than in matched normal tissues (Fig. 3a). Moreover, both CD147 and YIPF2 expression were markedly up-regulated at stage IV of HCC (Fig. 3b). Survival analysis showed that a higher expression of CD147 was significantly associated with shorter disease-free survival (DFS) and overall survival (OS), whereas YIPF2 expression was slightly associated with HCC survival (Fig. 3c, d). Interestingly, there were significant correlations between the expression of CD147 and YIPF2 both in HCC and in liver tissues (*p* < 0.01, *R* = 0.29–0.78) (Fig. 3e). These results indicated that YIPF2 co-expressed with CD147 is a predictor for HCC survival. Further protein interactive network analysis retrieved a few sorting-associated proteins (e.g., ATP6V1F) for CD147. In contrast, quite a number of proteins, which involved in vesicular trafficking (e.g., TMED1, KDELR1) or ER-Golgi transport (e.g., COPE, ZFPL1, PRKCSH), were enriched for YIPF2 (Fig. 3f). These data suggested that YIPF2 possibly mediates the trafficking and/or maturation process of CD147.

As CD147 and MMP were highly expressed/secreted in HepG2 and 7721 cells (Supplementary Fig. 3), we focused on these two cells in the remained of this study.

### YIPF2 mediates the mature processing of CD147 in ER-Golgi network

**Fig. 4:**
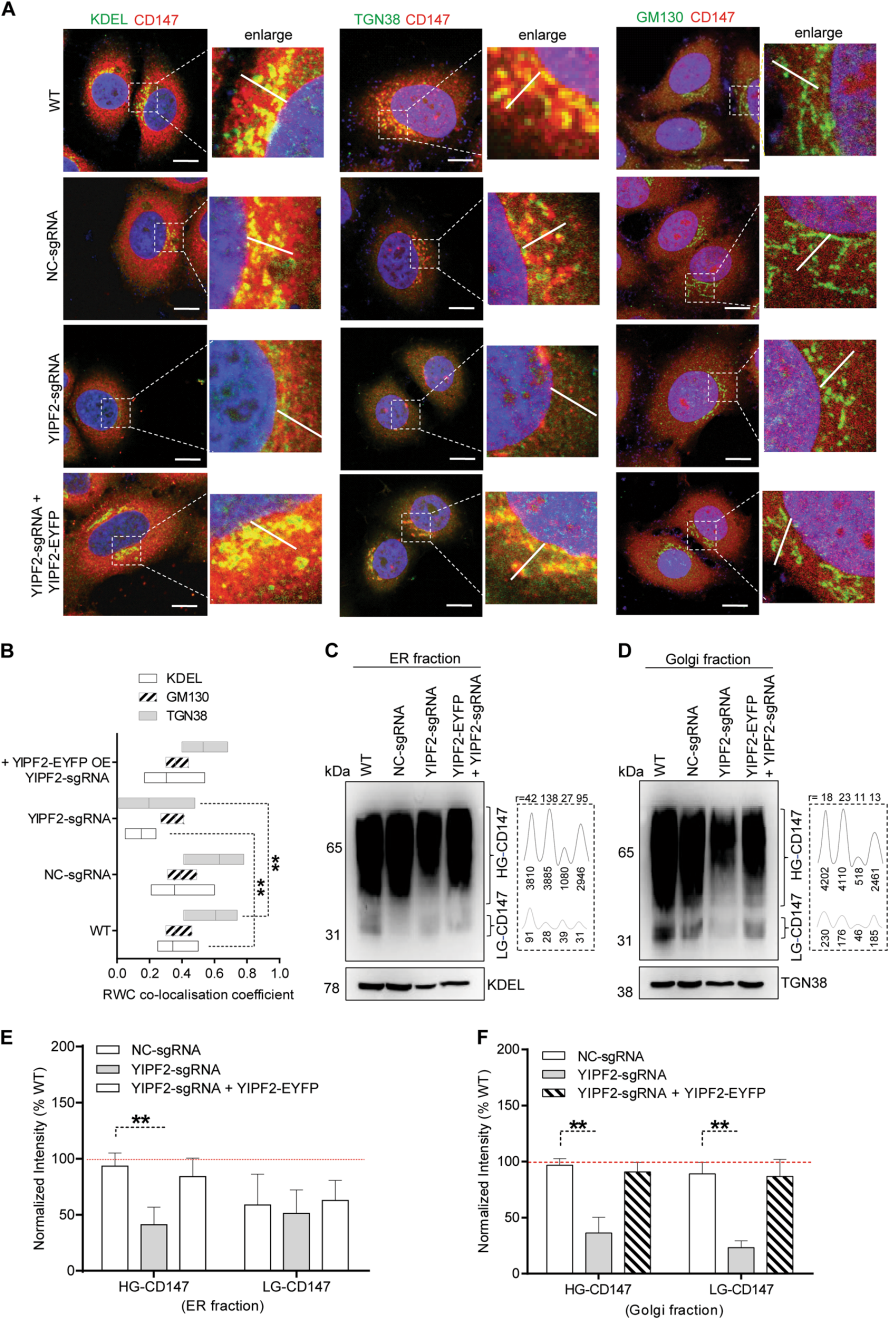
YIPF2 mediates the maturation processing of CD147 via the ER-Golgi trafficking route. YIPF2 knock-down dissipated ER-/Gogli-localized CD147. YIPF2-KD HepG2 cells (NC-KD cells as controls. WT: non-transfected HepG2 cells) were PFA-fixed, samponi-permeabilized, and stained by Ab combinations: anti-CD147 pcAb together with anti-KDEL, anti-GM130 and anti-TGN38 Abs, respectively (both plus corresponding anti-rabbit/mouse Ab-fluorescence). **a** Representative confocal observations are shown in the merged model. Scale bar: 20 μm. **b** Rank weighted coefficient (RWC) co-localization values of CD147 with KDEL, GM130, and TGN38. YIPF2 knock-down decreased the CD147 glycosylation level. Both HG- and LG-CD147 were blotted from the ER (**c**) and Golgi (**d**) fractions of treated HepG2 cells using H18 Ab. CD147 blotting (left) and quantitative scans of CD147 blots (right) by Image J software are shown. r values refer to HG/LG ratios. Numbers below indicate corresponding areas of CD147 peaks. Representative blot results from three independent experiments are shown (**c**, **d**), and corresponding quantitative data were analyzed (**e**, **f**). Statistically significant differences compared with NC-KD cells are shown: *n* = 3, **P < 0.01

To ascertain whether YIPF2 functions in CD147 maturation process, YIPF2 stable knock-down (YIPF2-KD) HepG2 cells were used to check CD147 retention in membranous organelles. Confocal imaging showed that YIPF2-KD significantly dissipated ER- and Golgi-localized CD147, and such dissipation was rescued by YIPF2 overexpression (Fig. 4a, b; Supplementary Figs. 5a, 5b, 6). Notably, overexpression of other YIPF family members also rescued this dissipation in YIPF2-KD cells (Supplementary Figs. 5c, 7). YIPF2-KD induced CD147 dissipation on ER and Golgi implies that the glycosylation of CD147 might be affected. Therefore, we investigated the glycosylation level of CD147 in ER- and Golgi-fractions of HepG2 cells. Western blotting showed that both HG-CD147 and LG-CD147 were markedly reduced in YIPF2-KD HepG2 cells, and YIPF2 overexpression can rescue this reduction (Fig. 4c–f). Similar confocal and Western blot results were obtained in 7721 cells (Supplementary Figs. 8, 9). These results suggested that YIPF2 can mediate the mature processing of CD147 via ER-Golgi trafficking route.

### YIPF2 controls the endocytosis and recycling of CD147

**Fig. 5:**
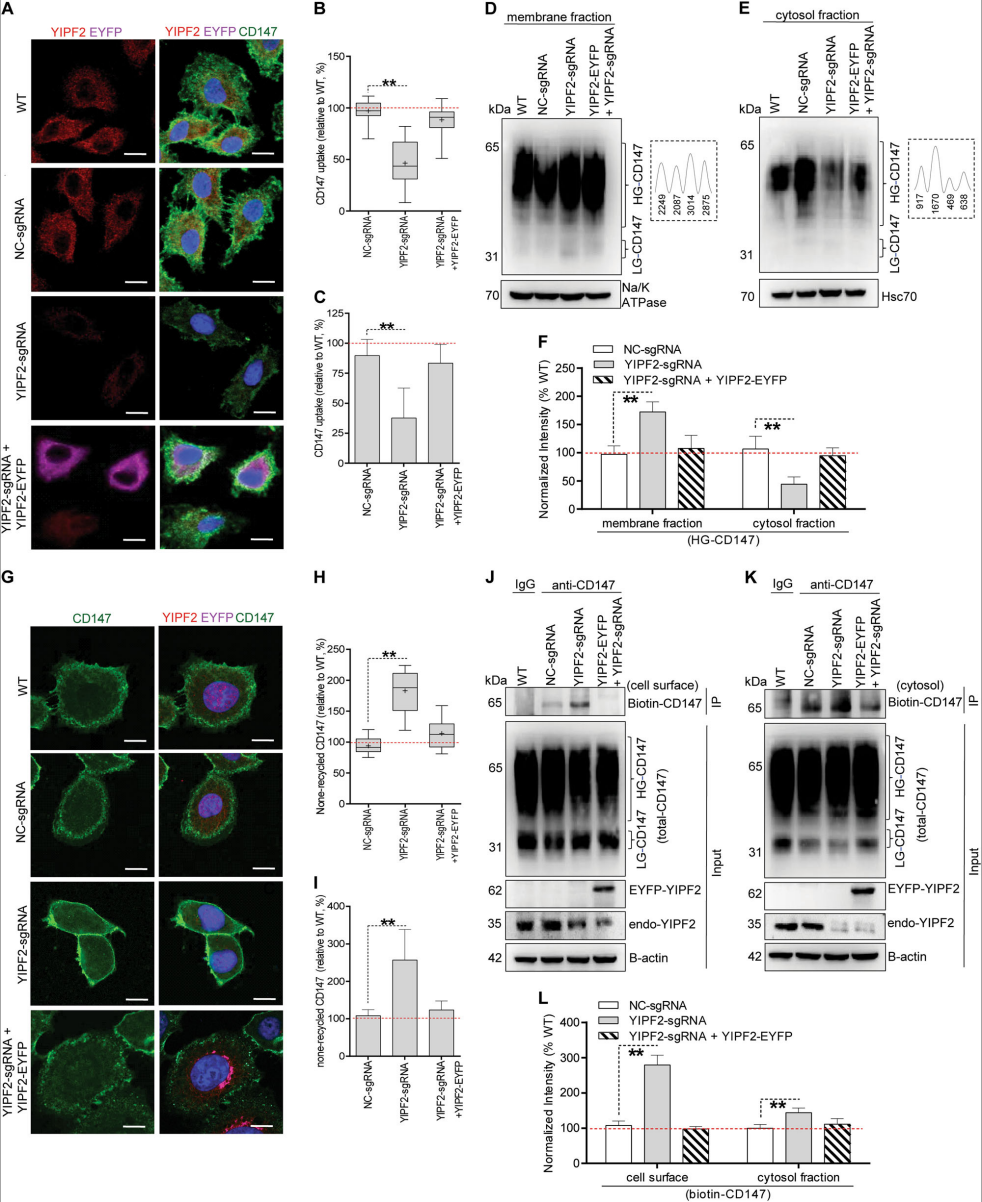
YIPF2 controls the endocytosis and recycling of CD147. YIPF2 knock-down reduced CD147 uptake. YIPF2-KD HepG2 cells (NC-KD cells as control) were transfected with the YIPF2/pdEYFP plasmid, then incubated with the H18Ab-AF488 complex at 37 °C for uptake, quickly rinsed, fixed, and Ab-stained (anti-YIPF2 to stain endogenous protein, anti-GFP to stain exogenous protein), and visualized via a confocal microscope. **a** Representative observations are shown. Scale bar: 20 μm. **b** Box-and-whiskers plots depict the uptake of the H18Ab-AF488 complex in cell populations. *n* = 60. **c** After cell surface digestion, the uptake of the H18Ab-AF488 complex in cells was quantified by flow cytometry. YIPF2 knock-down impaired glycosylated CD147 trafficking. HG-CD147 were blotted from the membrane (**d**) and the cytosol fraction (**e**) of treated HepG2 cells using H18 Ab. Quantitative scanning of CD147 blots are shown in the right dotted line box. YIPF2 knock-down decreased CD147 recycling. After removing the surface-bound H18Ab-AF488 complex, cells were removed at 37 °C for 30 min and the recycled complex on the cell surface was again removed. The no-recycling complex (i.e., intracellular residual portion) was visualized via a confocal microscope. **g** Representative observations are shown. Scale bar: 20 μm. **h** Box-and-whiskers plots depict the no-recycled CD147 pool in different cell populations. **i** The no-recycling H18Ab-AF488 complex inside HepG2 cells was quantified by flow cytometry. The uptake or no-recycled CD147 in untreated cells (WT) was set as 100% for comparison. Significant differences compared with NC-KD cells are shown: *n* = 3, **p < 0.01. Western blot determined the surface-resident biontin-CD147 (**j**) and intracellular non-recycled biotin-CD147 (**k**) after cell-surface biotinylation and anti-CD147 immunoprecipitation as described in ‘Materials and methods’. Representative blot results from three independent experiments are shown (**d**, **e**, **j**, **k**), protein bands were quantified by Image J software, and corresponding quantitative data were analyzed (**f**, **l**). Statistically significant differences compared with NC-KD cells are shown: *n* = 3, **p < 0.01

Since abnormal glycosylation of proteins always arise from altered intracellular transport^29–31^, we then examined whether CD147 trafficking was affected by YIPF2 interference. Confocal imaging showed that YIPF2-KD significantly reduced CD147 uptake in HepG2 cells. Conversely, YIPF2 overexpression reversed the reduced uptake of CD147 in YIPF2-KD cells (Fig. 5a, b). This action of YIPF2 on CD147 uptake was confirmed by flow cytometry results (Fig. 5c). Coupled with these observations, Western blotting showed that YIPF2-KD increased membrane-resident CD147 level but decreased cytoplasmic CD147 pool in HepG2 cells (Fig. 5d–f). Similar results were obtained in 7721 cells (Supplementary Fig. 8a, c, d, j).

As bioinformatics analysis predicted YIPF2 being a Rab-bound protein, and Rab regulate the endocytic recycling pathway by different modes^30,32^, we next examined the recycling of CD147 after YIPF2 intervention. Confocal and flow cytometry results showed that YIPF2-KD significantly increased surface-resident CD147 pool, and the increment was eliminated by YIPF2 overexpression (Fig. 5g–i). Notably, co-IP checking the dynamic change of CD147 in HepG2 cells showed that YIPF2-KD markedly increased biotinlated CD147 on cell surface, and significantly accumulated biotinlated CD147 in cytosol fraction (residual pool after recycling) (Fig. 5j–l). Similar results were obtained in 7721 cells (Supplementary Fig. 8g, h, l). These data suggested that YIPF2 expression dictated the endocytic recycling of CD147 in HCC cells, implying its traffic-control function possibly through Rab GTPase regulation.

### YIPF2 activates and recruits Rab5/22a to ER-Golgi network

**Fig. 6:**
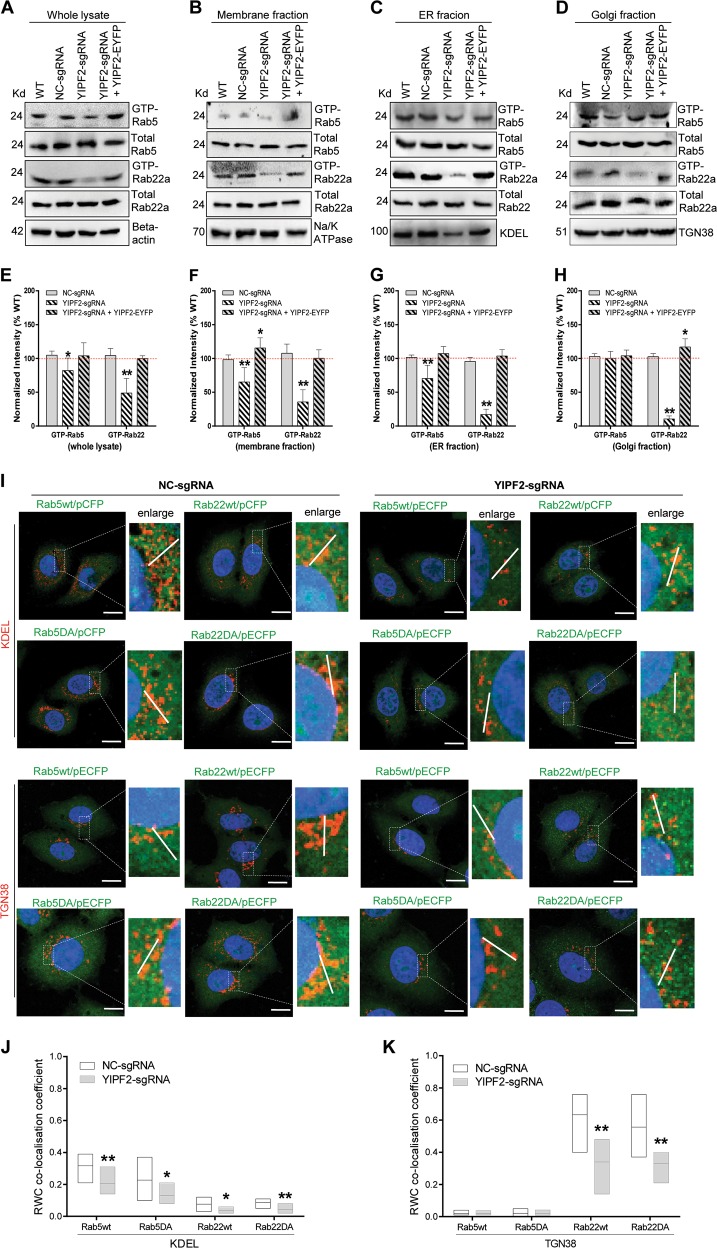
YIPF2 activates and recruits Rab5/22a to the ER-Golgi network. **a**–**d** YIPF2 knock-down decreased the activation of Rab5/22a. YIPF2-KD HepG2 cells (NC-KD cells as controls) when transfected with the YIPF2/pdEYFP plasmid and continued growth for 32 h. The cells were then serum-starved, lysed to extract different fractions, and incubated with GST-EEA or -Rabenosyn-5 beads. The activated Rab GTPases in the pull-down precipitate were blotted with corresponding Abs. Representative blot results from three independent experiments are shown. Activation of Rab GTPases was quantified by Image J software and shown in (**e**–**h**). Statistically significant differences compared with un-transfected cells (wt) are shown: *n* = 3, **p < 0.01, *p < 0.05. **i** YIPF2 knock-down dissipated the ER-/Gogli-localized Rab5/22a. Confocal imaging of the co-localization of wt- or DA-Rab5/22a with the ER marker (KDEL) and Golgi marker (TGN38). HepG2 cells were PFA-fixed, samponi-permeabilized, and stained with different Ab combinations: anti-GFP pcAb (plus anti-rabbit-AF488, green color) together with anti-KDEL Ab or anti-TGN38 Ab (both plus anti-mouse Ab-Alexa Fluor594, red color). Representative observations are shown. Scale bar: 20 μm. **j**, **k** Box-and-whiskers plots depict the co-localization variation of Rab5/22a with KDEL or TGN38 in cell populations. The rank weighted coefficient (RWC) values were quantified as described in ‘Materials and methods’. Significant differences compared with NC-sgRNA transfection are shown: *n* = 3, **p < 0.01, *p *<* 0.05

As Rab5 and Rab22a dominate the sorting and recycling of CIE proteins^30,31,33^, we checked whether altered YIPF2 expression can affect their activation. GST pull-down results showed that YIPF2-KD significantly attenuated the activations of Rab5 and Rab22a, and such attenuation was rescued by YIPF2 overexpression in HepG2 cells (Fig. 6a–e). Given that Rab GTPases mediate protein transport highly relay on their tethering to membranous structures^29,34^, we speculated that YIPF2-KD restrained the recruitment and activation of Rab5/Rab22a through ER-Golgi networks. Confocal imaging showed that YIPF2-KD markedly weakened ER-localized Rab5wt and Rab5DA, whereas ER-localizations of wt-Rab22a and DA-Rab22a were not affected in HepG2 cells (Fig. 6i, j. Supplementary Fig. 10). In contrast, YIPF2-KD only weakened the localizations of wt-Rab22a and DA-Rab22a in Golgi (Fig. 6i–k. Supplementary Fig. 10). Further determining the membrane-associated Rab showed that YIPF2-KD caused Rab22a activation to decrease in all checked membranous fractions (Fig. 6b–h). However, no Rab5 activation reduced in Golgi was detected after YIPF2-KD (Fig. 6d–h). Together, these data suggested that Rab5/22a recruitment and activation at ER-Golgi network depend on YIPF2 protein level.

### YIPF2 modulates CD147-mediated malignant phenotypes

**Fig. 7:**
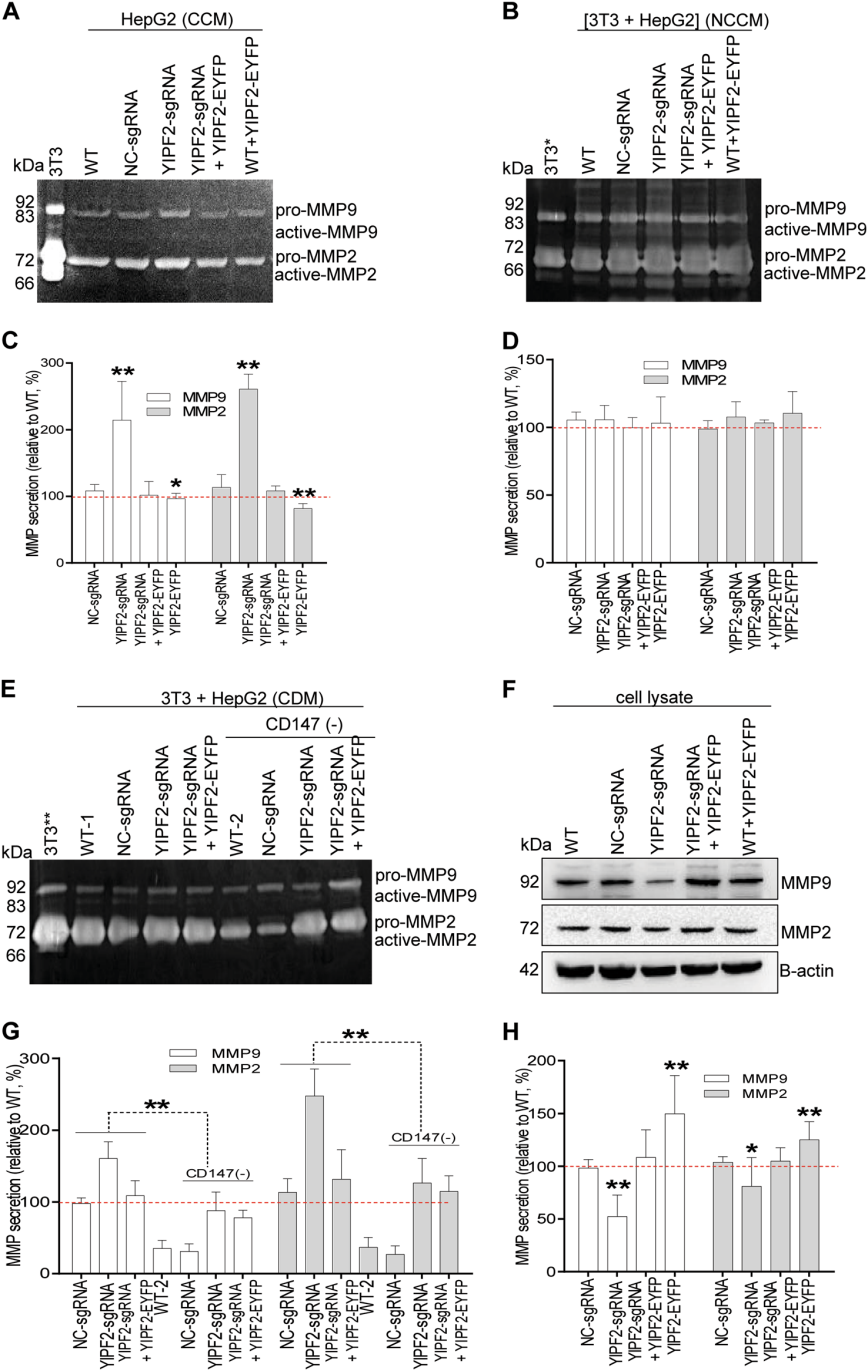
YIPF2 knock-down increased MMP secretion. YIPF2-KD HepG2 cells were transfected with the YIPF2/pEYFP plasmid. **a**, **b**, **e** Forty-eight hours after transfection, secreted MMPs activity in concentrated medium (CCM) from mono-culture (**a**), in non-concentrated medium (NCCM) from co-culture with 3T3 cells (**b**), and in conditioned medium (CDM, pre-depleted CD147 by H18 Ab) from 3T3 cells (**e**) were detected by gelatin zymography. **f** Western blot determined the endogenous MMP in treated cells. Representative results from three independent experiments are shown (**a**, **b**, **e**, **f**), protein bands were quantified by Image J software, and corresponding quantitative data were analyzed (**c**, **d**, **g**, **h**). Statistically significant differences compared with non-treated cells (WT) are shown: *n* = 3, **p < 0.01, *p *<* 0.05

MMP-mediated extracellular matrix (ECM) degradation is essential to HCC invasion, and MMP secretion is trigged by CD147-mediated intercellular interaction^5–8^. Gelatin zymography results showed that YIPF2-KD significantly increased MMP2 and MMP9 secretion in HepG2 cells, but such an increase was rescued by YIPF2 overexpression (Fig. 7a–c). Although YIPF2-KD induced MMP secretion increase were not detected in co-culture medium (Fig. 7b–d), the promotion phenomena reappeared when HepG2 medium was added to 3T3 cells (Fig. 7e–g). Notably, when CD147 was depleted from cell medium, YIPF2-KD or overexpression induced MMP2/9 secretion from 3T3 cells were attenuated (Fig. 7e–g). Additionally, Western blotting showed that YIPF2-KD significantly reduced endogenous MMP2 and MMP9 levels in HepG2 cells, and such a reduction was rescued by YIPF2 overexpression (Fig. 7f–h). Similar results were obtained in 7721 cells (Supplementary Fig. 11). These results suggested that YIPF2-KD induced CD147 delivery to HCC cell-surface and release to extracellular medium, which promoted MMP secretion.

**Fig. 8:**
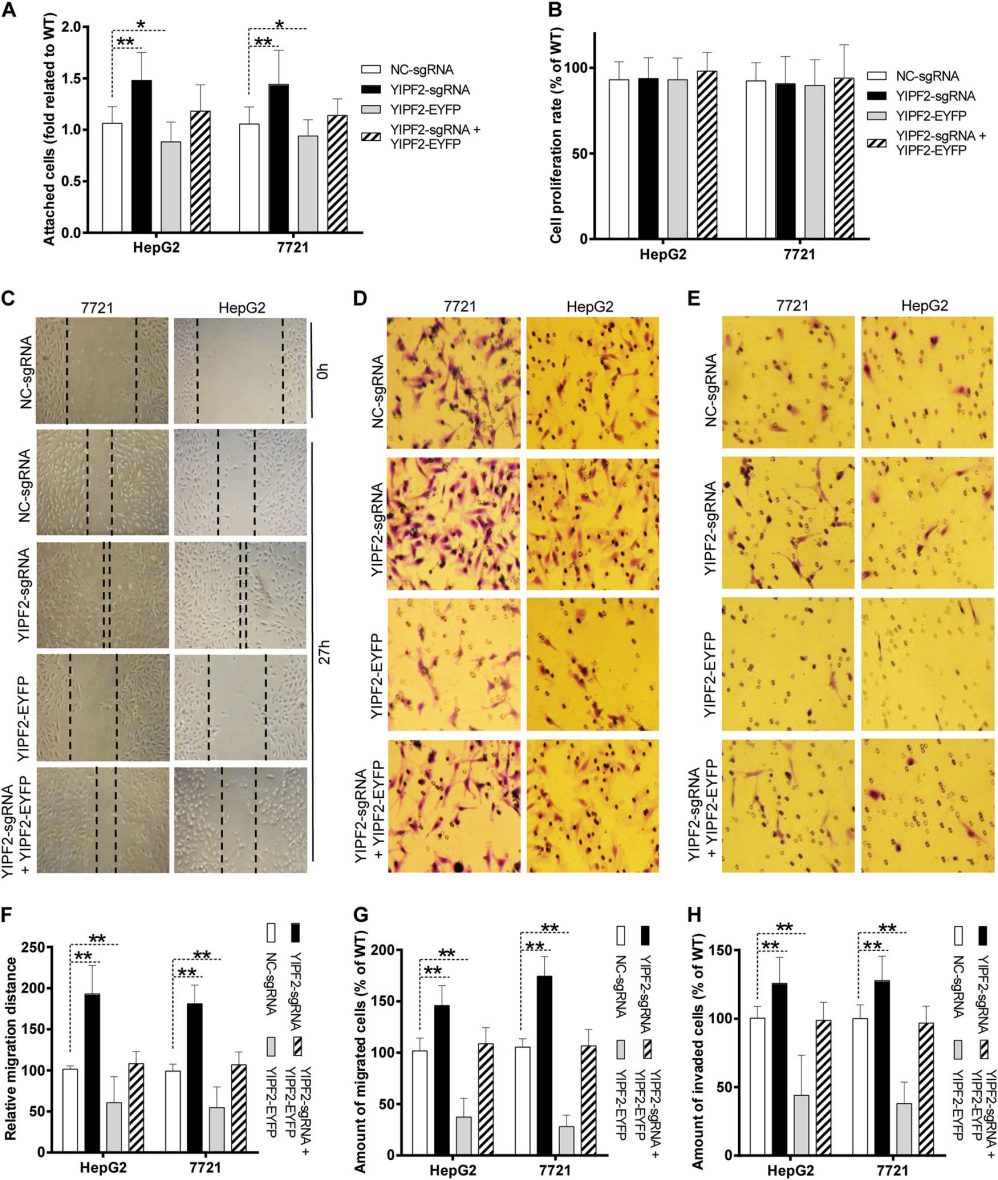
YIPF2 knock-down enhanced the adhesion, motility, migration, and the invasion potential of HCC cells. YIPF2-KD HepG2 and 7721 cells were respectively transfected with the YIPF2/pEYFP plasmid. **a** Cell adhesion. Forty-eight hours after transfection, cells were re-seeded and equal numbers of cells were added to Matrigel-coated wells for 1 h. Attached cells were crystal-violet-stained, lysed, and detected. **b** Cell proliferation. Sixteen hours after transfection, cells were re-seeded and equal numbers of cells were cultured for 32 h. Numbers of viable cells were measured using the CCK-8 kit. **c** Cell motility. Cells were cultured until confluent. After scratching, cells were cultured with medium containing 10% FBS. Photomicrographs at 10× magnifications were taken at 24 h. **d** Cell migration. Twenty-four hours after transfection, cells were re-seeded and equal numbers of cells were added into the upper chamber. Twenty-four hours later, the cells migrating through the filter were crystal-violet-stained and counted. **e** Cell invasion. Similar procedures were performed as described in (**d**) except for adding cells into Matrigel-coated upper chamber and supplying HGF in the low chamber. Representative results from three independent experiments are shown in (**c**–**e**), and corresponding quantitative data were summarized in (**f**–**h**). Statis-tically significant differences compared with NC groups are shown: *n* = 3, **p < 0.01, *p *<* 0.05. WT: non-treated cells. NC-sgRNA: negative control

As YIPF2 is critical for CD147 trafficking, we further speculated that YIPF2-KD highly induced CD147 presenting on cell-surface and more extensively promoted the malignant phenotypes of HCC cells. Our results showed that YIPF2-KD markedly increased the adhesion of HepG2 and 7721 cells to Matrigel, but this potential was reversed by YIPF2 overexpression (Fig. 8a). Since altered ECM adhesion and MMPs secretion are correlated with modified motility, migration, and invasion capabilities of cancer cells^32^, we then checked these behaviors. As expected, YIPF2-KD markedly enhanced the motility, migration, and invasion of HepG2 and 7721 cells. Conversely, YIPF2 overexpression remarkably restrained these characteristics (Fig. 8c–h). We also checked the contribution of cell proliferation to these characteristics, and no cell proliferation was affected by YIPF2 intervention (Fig. 8b). Together, these results suggested that YIPF2-KD enhanced the adhesion, motility, migration, and invasion behaviors of HCC cells.

## Discussion

The function of CD147 as a driver of malignant neoplasms strongly depends on its cell surface presentation. Although several binders were reported to form complexes with CD147^19–27,35,36^, none appear to govern CD147 trafficking in cancer cells. MAPPIT offers the combined advantages of optimal physiological context, separated interactor-effector design, and stimulation-responsive screening to determine dynamic interactions during antigen trafficking^28,37^. We used the advantages of MAPPIT, screened and identified YIPF2 as a new binder to CD147 with endocytic recycling regulation function.

The name of YIPF refers to the Ypt (yeast Rab GTPase)-interacting protein. Previous studies showed that YIPF proteins have five transmembrane segments with N-terminal exposed to the cytoplasm and C-terminal exposed to the lumen of secretory endomembranes^38^. Because YIPF proteins form complexes with each other and are found in ER, Golgi, and COPII vesicles, they were predicted to play roles in maintaining ER-Golgi morphology and/or in vesicle transport between organelles^38–40^. We found here that YIPF2 is mainly localized in ER and trans-Golgi of HCC cells (Fig. 2b). Unlike wide localization across endomembrane in YIPF2-overexpressed cells^38^, we hardly viewed endosome-localized YIPF2 in HCC cells unless Rab5/22 was also stained for co-localization analysis (Supplementary Fig. 4b). We found that YIPF2-KD dissipated ER- and trans-Golgi-localized CD147, and these dissipations coincided with ER-Golgi fragmentation and dispersion in HCC cells (Fig. 4a, b). This result is in contrasted with a recent study that YIPF2-KD induced no Golgi structure alteration but delayed Golgi reassembly in colon cancer cells^41^. The reason why YIPF3 and YIPF4 overexpression could rescue the dissipated ER-Golgi-localized CD147 in YIPF2-KD cells is not known (Supplementary Fig. 7). Although YIPF3 and YIPF4 are cis-Golgi-localized YIPFs, they always act together with trans-Golgi-localized YIPF2 on regulating endomembrane dynamics. As previous studies showed YIPFs co-precipitates with the components of cellular trafficking machineries and synergistically play roles in cargo trafficking^39,42^, it is not surprising that YIPF3/4 overexpression recovered both impaired Golgi morphology and distributed secretory pathway in HCC cells.

**Fig. 9:**
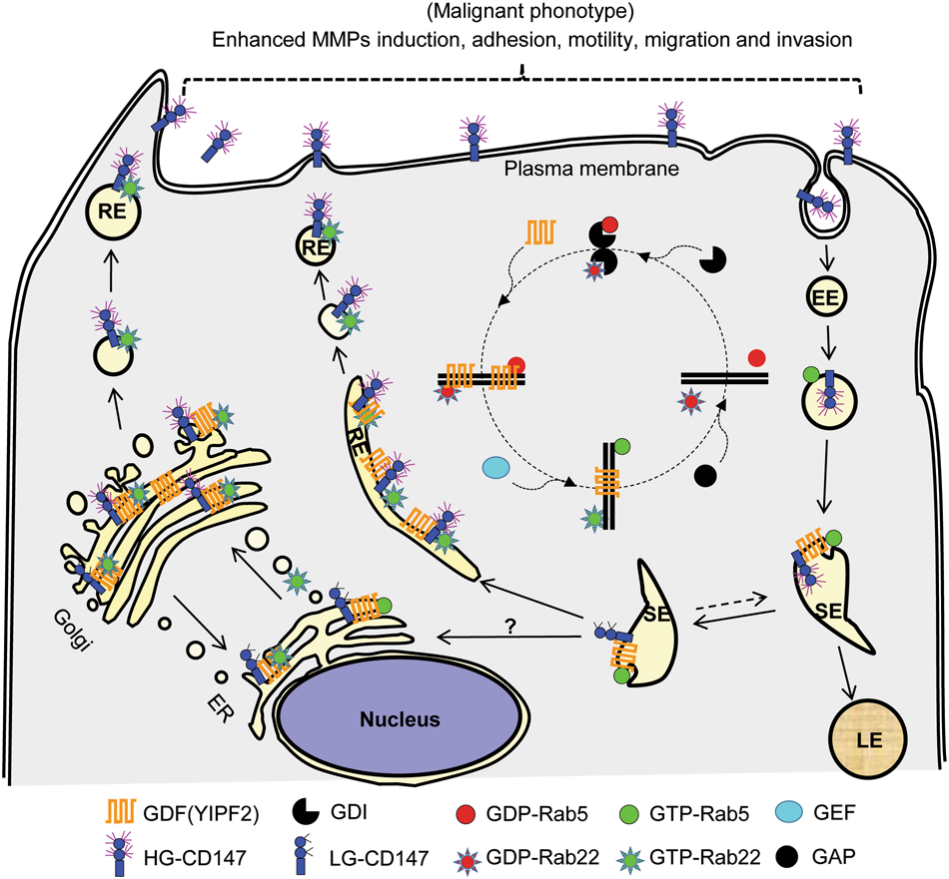
Schematic model of YIPF2 functions as a Rab-GDF on CD147 trafficking. Current results of YIPF2-mediated CD147 sorting, mature glycosylation, and endocytic recycling, which mainly involve dynamic activation and recruitment of Rab5/Rab22a to distinct membranous organelles, are summarized together with previous knowledge^19,45,59^. See Discussion for details. GDI GDP dissociation inhibitor, GDF GDI displacement factor, GEF guanine nucleotide exchange factor, GAP GTPase accelerating protein, Golgi, Golgi apparatus, ER endoplasmic reticulum, LE late endosome, RE recycling endosome, EE early endosome, SE sorting endosome

Being an N-linked glycosylated protein, immature CD147 is preliminary glycosylated with 1 glycan in ER, and then transported to Golgi for further modification by glycosyltransferases including GnT-III, GnT-IV, GnT-V, and FuT-8^6^. As glycosyltransferases cannot work without integrated ER/Golgi morphology and YIPF2 interference hinders intracellular glycan synthesis^41^, it is logical that LG- and HG-CD147 are reduced in ER-Golgi fractions from YIPF2-KD HCC cells (Fig. 4c–f). Some researchers may argue that Golgi is the apparatus wherein O-linked glycan chains are synthesized, how could the N-linked glycosylated CD147 be affected by YIPF2-induced Golgi morphology change? One explanation is that N-glycosylation modifications occur in both ER and Golgi. Another may be because Yif1p associates with Yip1p (yeast homolog of YIPF6) and plays role in membrane fusion within Golgi^43^. Considering that branched N-glycans are catalyzed by GnT family glycosyltransferases which mainly located in Golgi^6^, and CD147 is a target of GnT-V^44^, it is probable that YIPF2-KD attenuated YIPF2–YIPF6 complex, causing delayed recycle of glycosyltransferases or incomplete vesicle fusion in Golgi, which impaired the glycosylation maturation of CD147 in ER-Golgi compartments (Fig. 9).

Rab GTPases regulate the trafficking of membrane proteins^30,31,34^. Previous studies showed Rab5 activation boosted the early steps of CD147 uptake, and Rab22a activation accelerated CD147 recycling to cell surface^33,45^. We found that Rab5 and Rab22a activation were reduced by YIPF2-KD in HCC cells, and such reductions were severe in membrane fractions (Fig. 6a–f). This result is logical, because the yeast Yif1P is Rab-bound protein, and Rab is activated only when it was periodically recruited to membranes^46^. One interesting finding is that YIPF2-KD induced distinct inactivation and dissipation of Rab GTPases (Fig. 6). Although Rab5 can work together with other GTPases to direct CIE cargos (such as CD147) transport to recycling endosomes, Rab22a is key driver for CIE sorting machinery^33^. The more inactivation of Rab22a than Rab5 by YIPF2-KD (Fig. 6a–h), the more recycling of CD147 was impaired, suggesting YIPF2 mainly functions on the late sorting steps of CD147.

What is the role played by YIPF2 on CD147 transport? Because yeast YIP1 family are counterparts to Rab-GDI (guanine nucleotide dissociation inhibitor) and they complex together to establishing the fusion of ER/Golgi vesicles at the time of budding^40,46^, the best possibility is that YIPF2 serves as an acceptor which aids in recruiting Rab from the cytosol onto endosomes or ER-Golgi, enabling Rab to be correctly localized, activated, and used for many rounds of transport. Although we did not check whether YIPF2 affects other Rab GTPases, one study showed that Yif1p can bind with several Rab GTPases^46^. Notably, these Yif1p-bound Rabs are required for vesicle transport in endocytic pathway (YPT10, YPT52), secretory pathway (YPT31, YPT32, SEC4), endosome docking or fusion (VPS21, YPT7), ER-Golgi trafficking (YPT1), and post-Golgi secretion to cell surface (YPT6, SEC4)^46^. Because Rab5 and Rab22a activation trigger CD147 endocytic recycling^33,45,47^ and YIPF2 determines their activation and localization (Fig. 6), we propose a model: Rab5 and Rab22a bind to GDI in cytosol, which keeps them in inactive state. YIPF2 acts as a GDF (GDI-displacement factor) to catalyze the dissociation of Rab-GDI complexes, and to enable transfer of Rab5/22a from GDI onto distinct endosomes or ER-Golgi membranes. Activated Rab5 and Rab22a localize to distinct compartments and regulate CD147 transport from endocytosis, sorting and recycling to ER-Golgi trafficing/secretion (Fig. 9).

In summary, we identified YIPF2 as a Rab-GDF regulating CD147 endocytic recycling, and the surface level of CD147 is controlled by YIPF2 in HCC cells. Our results reveal a YIPF2-controlled ER-Golgi trafficking signature promotes CD147-medated malignant phenotype in HCC.

## Materials and methods

### Cell cultures, plasmids, antibodies, and chemicals

Materials are listed in Supplementary ‘detailed material and methods’.

### Construction of retroviral MSP-cDNA library

The process was performed as previously described^10^. Briefly, total RNA was drawn from two human liver cancer tissues. The MSP-enriched cDNA gene pool was PCR-amplified by using primers (Supplementary Table 1), digested with EcoR I/Not I, and cloned into pBG1 plasmid. The recombinant plasmids were electro-transformed into *E*. *coli* JM109 cells and culture-amplified. MSP-cDNA library plasmids were transfected into Plat-E cells. One flask of cells was transfected with retroviral cDNA library and EGFP reporter plasmid. The culture supernatant containing retroviruses was harvested; viral morphology and titer were determined by electron microscopy and flow cytometry, respectively.

### MAPPIT screening and analysis

MAPPIT were performed as previously described^28^. Briefly, EpoR/LR-F3/CD147EP-expressed HEK293-16 cells (1.5 × 10^6^) were cultured in medium containing Epo (5 ng/ml) with puromycin (0.5, 1, 1.5, and 2 μg/ml). The background was determined based on the number of colonies (1 or 2 per flask) that survived under double-selection, but without infection by the retroviral cDNA library. chimeric receptor-expressed Cells were infected with the retroviral MSP-cDNA library, and then cultured under double selection. Total RNA was drawn from the surviving colonies, and genes of potential binders were PCR-amplified and cloned into pMD18-T vector. For analytical assays, HEK293T cells (3 × 10^5^) were co-transfected with bait plasmid (pSEL1/CD147EP or pSEL1/CD147IP) and prey plasmid (pMG1/YIPF2) together with reporter (pXP2d2-rPAP1-luci) plasmid. After overnight growth, cells were reseeded in plates for EPO (5 ng/mL, 24 h) stimulation or not. Survived cells were lysed followed by adding luciferase substrate for activity analysis.

### Gene stable knock-down and transient overexpression

The CRISPR/Cas9 lentivirus system (from Addgene) was utilized to produce YIPF2-KD HCC cells. Three independent YIPF2-targeting sgRNA (sgRNA#1: CACCGCGAGGAGGCCACTAATCTTC, sgRNA#2: CACCGGACCTTCCATGAATTCGAGG, and sgRNA#3: CACCGTAGTGGCCTCCTCGAATTCA) were generated as previously described^48^. YIPF2/3/4pdEYFP and Rab5/Rab22a/pECFP-C1 plasmids were respectively transfected in HCC cells for gene overexpression.

### Uptake measurement and ER/Golgi co-localization analysis

CD147 uptake and binding were measured as previously described^48–51^. Incubation conditions for the H18Ab complex were optimized (H18Ab, 1:500, anti-mouse AF488, 1:1000, 15 min) in HCC cells. YIPF2-interference cells were PFA-fixed, triton-permeabilized, and stained by anti-KDEL/TGN38/GM130 Abs (1:100) together with anti-CD147 pcAb (1:2000) or with anti-GFP Ab (1:500, recognized Rab5/22a/pECFP), then stained by corresponding fluorescence-labeled 2^nd^ Ab. Images were analyzed with Image J software, and co-localization was calculated as the Rank Weighted Coefficient (RWC)^52^. In confocal imaging process, 60 cells were observed in each transfection. In flow cytometry measurements, 10,000 cells were counted per sample.

### Cell surface biotinylation and antigen recycling assay

CD147 recycling was determined as previously described^45,53,54^. Briefly, cells were labeled with biotin (0.2 mg/ml) at 4 °C, washed with glycine (0.1 M, PH8.0), and transferred for uptake at 37 °C. At 15 min, cells were washed with PBS, and biotin was removed from cell-surface by incubation with 50 mM DTT. After continue-incubating at 37 °C for 15 min, cells were returned to the ice and biotin was removed from recycled proteins by a second DTT reduction. Cells were RIPA-lysed or further fractioned by utilizing cell fraction kits. Supernatants were corrected to equivalent protein concentration and levels of biotinylated CD147 (cell-surface portion vs intracellular non-recycled portion) were determined by co-IP.

### Rab GTPase activation assay

Rab GTPase activation were performed as previously described^55,56^. GST-EEA1 (residues 36–91) immobilized beads (for Rab22a activation) and GST-Rabaptin-5 (residues 739-e) immobilized beads (for Rab5 activation) were produced, respectively. After subjecting the collected pellets to SDS-PAGE, the GTP-bound Rab5 and Rab22a in samples were determined by Western blotting.

### Cell adhesion, proliferation, migration, motility, and invasion assays

The processes were performed as previously described^10,57^. In Transwell motility and invasion assays, the lower chamber was filled with 10% FBS medium containing HGF (20 ng/ml).

### Cell fractionation, Western blot, co-IP, and Gelatin zymography assays

The process was similar to that as previously described^10,57^. Concentrated supernant from single-cultures were loaded for Gelatin zymography assay.

### Bioinformatics and statistical analysis

Microarray mining analysis were performed based on GEPIA (Gene Expression Profiling Interactive Analysis) database (http://gepia.cancer-pku.cn/)^58^. The ANOVA differential method was used for tumor (T) vs paired normal (N) samples. Confocal microscopy, flow cytometry, and Western blotting data were derived from three independent experiments. All data drawn from the experiments were analyzed using GraphPad Prism 5 software and are described as the mean ± SEM values.

## Supplementary information

Detailed Material and Method

PCR primers used in this study

Construction and identification of a retrovirus MSP-cDNA library

Selection of positive clones after MAPPIT screening

Identifying the MMP secretion and CD147 expression in HCC cells

Subcellular localization of YIPF2 and CD147

Stable knock-down and transient overexpression of YIPF2

Co-localization profiles of CD147 with ER/Golgi markers

RWC values of CD147 co-localized with ER/Golgi markers after YIPF3 (a) and YIPF4 (b) overexpression in YIPF2-KD HepG2 cells

YIPF2 regulates the endocytosis, ER-Golgi trafficking, glycosylation, and recycling of CD147

YIPF2 knock-down dissipated ER-/Gogli-localized CD147

Co-localization profiles of Rab5/Rab22a with ER/Golgi markers

YIPF2 knock-down increased MMP secretion in 7721 cells
