# Supplementary material for: YIPF2 is a novel Rab-GDF that enhances HCC malignant phenotypes by facilitating CD147 endocytic recycle
Source: Cell Death Dis. 2019 Jun 12;10(6):462. doi: 10.1038/s41419-019-1709-8 (PMC6561952; doi:10.1038/s41419-019-1709-8)
Supplement: Supplementary file 1 — Detailed Material and Method [file 41419_2019_1709_MOESM1_ESM.docx]

**Supplemental: Detailed Material and Methods**

**Cell cultures, plasmids, antibodies, and chemicals**

Human normal liver cell 7702, HCC cells 7721, HepG2, Hep3B, MHCC-97H, Huh7, mouse fibroblast 3T3, and human embryonic kidney 293T cell obtained from Type Culture Collection of Chinese Academy of Sciences (China), were routinely cultured in DMEM containing 10% (v/v) fetal bovine serum. Plat-E cells were kindly supplied by Prof. T Kitamura (Tokyo University)[^1^](#_ENREF_1). EpoR/LR-F3/CD147EP stable-expressed HEK293-16 cells were once produced[^2^](#_ENREF_2).

YIPF2, YIPF3, and YIPF4/pdEYFP plasmids were kindly supplied by Prof. Vytaute Starkuviene (Heidelberg University)[^3^](#_ENREF_3). EEA1, Rabaptin-5/pGEX-4T-3 plasmids, wild type Rab5(wt), Rab22a (wt), dominant active Rab5(Q79L), and Rab22a(Q64L)/pECFP-C1 plasmids were kindly supplied by Prof. Byung-Ha Oh (Korea Advanced Institute of Science and Technology)[^4^](#_ENREF_4). CD147EP (0-229) and CD147IP (229-269)/pEGFPN1 plasmids were previously constructed[^5^](#_ENREF_5). pCEL2f, pBG1, pSEL1, and pMG1 plasmids were utilized for the MAPPIT assay[^6^](#_ENREF_6). The lentiviral CRISPR/Cas9 system was kindly supplied by Prof. Zhang Feng (Broad Institute of MIT and Harvard)[^7^](#_ENREF_7).

Mouse anti-CD147 Ab (H18) was originally produced[^5^](#_ENREF_5)^,^[^8^](#_ENREF_8)^,^[^9^](#_ENREF_9). Rabbit anti-CD147 Ab (AB22048b), anti-YIPF2 Ab (SAB2108420), mouse anti-YIPF2 Ab (sc-398530), anti-KDEL Ab (sc-58774), anti-GM130 Ab (sc-55590), anti-TGN38 Ab (sc-166594), anti-Rab5 Ab (D160063), anti-Rab22a Ab (D160036), Rabbit anti-biotin Ab (D5A7), anti-Hsc70 Ab (sc-7298), anti-Na/K ATPase Ab (sc-71637), and anti-biotin-HRP Ab (sc-53179) were obtained from Santa Cruz. Mouse anti-GFP Ab (KM8009), rabbit anti-GFP Ab (D110008), mouse anti-MMP9 Ab (WL01580), anti-MMP2 Ab (P106506), anti-β-actin Ab (D190606), cell Counting Kit-8 (E606335), and cell fractionation (membrane/cytoplasm, ER, Golgi) isolation kits (BSP073, BB31454, BB31453) were obtained from Sangon Biotech and Bestbio. Goat anti-mouse Ab-Alexa Fluor488 (A-11001), anti-rabbit Ab-Alexa Fluor488 (A-11070), anti-rabbit Ab-Alexa Fluor594 (R37117), anti-mouse Ab-Alexa Fluor594 (R37121), Lipofectamine 2000 (11668027), Dynabeads proteinG (10003D), GSH Glutathione sepharose (G2879), Hochest33342 (62249), and BCA Protein Assay Kit (23225) were obtained from Life Technologies. The Luciferase assay kit (E1501) was from Promega. Matrigel (356234) was from BD Bioscience. EPO (#287-TC-500) and hygromycin B (#4089) were from R&D Systems. Gelatin (G1890), polybrene (H9268), puromycin (P7255), NHS-SS-biotin (21328), and the remaining fine-grade chemicals used throughout this study were all from Sigma.

**Gelatin zymography assay**

The process was similar to that previously described[^5^](#_ENREF_5)^,^[^9^](#_ENREF_9). For single-cultures, 24h cultured medium (serum-free) was collected from YIPF2-KD or -overexpressed HCC cells, and then 40 fold concentrated by using Amicon Ultra-4 10 k devices. Equivalent amounts of protein were, separately loaded in SDS-PAGE gel containing 1% gelatin, washed by 2.5% Triton, incubated with incubation buffer, and finally stained with 0.05% Coomassie blue. MMP-9 and MMP-2 activity was considered the unstained areas. For co-cultures, 3T3 cells and HCC cells were mix-seeded at a density ratio of 1:3 to co-culture and the MMP level change was calculated as described above. In some cases, the secreted CD147 antigen was deleted from the culture medium by H18 Ab immunoprecipitation for subsequent assays.

**Cell adhesion and proliferation**

The processes were similar to that previously described[^5^](#_ENREF_5)^,^[^9^](#_ENREF_9). Cell adhesion: YIPF2-KD or -overexpressed HCC cells (2×10^4^/well) in serum-free medium were added to Matrigel-coated 96-well plates and incubated for 1h. After removing the medium and non-attached cells, 0.1% crystal violet was added, tap-washed, air-dried, lysed with 5% SDS, and the absorbance was read at 540nm. Cell proliferation: equal densities of YIPF2-interferred HCC cells were inoculated in advance. After culture for 48 hours in 96-well plates, 10 μl of the CCK-8 solution was added in each well, the plate was incubated for 1h and the absorbance was measured at 450nm.

**Cell migration, motility, and invasion assay**

*In* *vitro* wound assay: YIPF2-KD or -overexpressed HCC cells were seeded in 12-well plates (23×10^4^ cells per well, coated with 0.6% gelatin,) in serum-free medium, and starved overnight. After scraping the cell monolayer with a sterile micropipette tip, medium with 10% FBS was added (t=0). Cells were photographed using a phase-contrast microscope at desired times, the remaining wounded area was measured using Image J software, and cell migration was calculated as percent wound closure of the scratched area at t=0.

Transwell motility and invasion assays: 5×10^4^ YIPF2-interferred HCC cells were starved overnight, harvested by trypsinization, and seeded wiht serum-free medium in Matrigel-coated or uncoated culture inserts (Transwell; 24-well plate with 8-μm pore size; Millipore). The lower chamber was filled with 10% FBS medium containing 20 ng/ml HGF. After 24-h incubation and the cells on the filter of the upper chamber were removed with a cotton swab, the cells on the underside were stained with crystal violet and counted under a microscope.

**Cell fractionation, Western blot, and co-IP**

The cytosol, membrane, ER and Golgi fractions of YIPF2-interferred HCC cells were extracted according to the instructions. The protein concentration of the samples was measured by a BCA kit, resolved by SDS-PAGE, blotted onto PVDF membranes, and then analyzed with different Abs diluted as following: mouse anti-CD147 Ab (H18, 1:1000), anti-MMP2 Ab (1:500), and anti-MMP9 Ab (1:500). Rabbit anti-YIPF2 Ab (1:1000), rabbit anti-GFP Ab (1:500), anti-B-actin Ab (1:2000), anti-Na/KATPase Ab (1:2000), anti-Hsc70 Ab (1:500), anti-KDEL Ab (1:500), anti-TGN38 Ab (1:500), anti-Rab5 Ab (1:500), anti-Rab22a Ab (1:500), and anti-biotin-HRP (1:500).

For immunoprecipitation, cells were lysed in IP lysis buffer followed by pre-clearing with protein A/G for 1 h. A cell lysate (200 μg total proteins) was incubated with anti-YIPF2 or H18 Abs (2 μg) overnight at 4°C, and then incubated with protein A/G for an additional hour. Beads were washed four times with IP lysis buffer before being transferred for Western blot.

**References**

1 Morita, S., Kojima, T. & Kitamura, T. Plat-E: an efficient and stable system for transient packaging of retroviruses. *Gene therapy* **7**, 1063-1066, doi:10.1038/sj.gt.3301206 (2000).

2 Zhao, P., Zhang, S. H., Jan, T., Li, Y. & Chen, Z. N. [Development of recombinant HEK293-16 cell strain expressing chimera receptor EpoR/LR-F3/HAb18GEF with site-specific integration expression system]. *Xi bao yu fen zi mian yi xue za zhi = Chinese journal of cellular and molecular immunology* **23**, 213-216 (2007).

3 Lisauskas, T. *et al.* Live-cell assays to identify regulators of ER-to-Golgi trafficking. *Traffic (Copenhagen, Denmark)* **13**, 416-432, doi:10.1111/j.1600-0854.2011.01318.x (2012).

4 Ku, B. *et al.* VipD of Legionella pneumophila targets activated Rab5 and Rab22 to interfere with endosomal trafficking in macrophages. *PLoS pathogens* **8**, e1003082, doi:10.1371/journal.ppat.1003082 (2012).

5 Zhao, P. *et al.* HAb18G/CD147 promotes cell motility by regulating annexin II-activated RhoA and Rac1 signaling pathways in hepatocellular carcinoma cells. *Hepatology (Baltimore, Md.)* **54**, 2012-2024, doi:10.1002/hep.24592 (2011).

6 Eyckerman, S. *et al.* Design and application of a cytokine-receptor-based interaction trap. *Nature cell biology* **3**, 1114-1119, doi:10.1038/ncb1201-1114 (2001).

7 Shalem, O. *et al.* Genome-scale CRISPR-Cas9 knockout screening in human cells. *Science (New York, N.Y.)* **343**, 84-87, doi:10.1126/science.1247005 (2014).

8 Xu, J. *et al.* A randomized controlled trial of Licartin for preventing hepatoma recurrence after liver transplantation. *Hepatology (Baltimore, Md.)* **45**, 269-276, doi:10.1002/hep.21465 (2007).

9 Zhao, P. *et al.* Annexin II promotes invasion and migration of human hepatocellular carcinoma cells in vitro via its interaction with HAb18G/CD147. *Cancer science* **101**, 387-395, doi:10.1111/j.1349-7006.2009.01420.x (2010).

**Supplemental Tab. 1 PCR primers used in this study**

| Primers | Sequence |
| --- | --- |
| MSPs-cDNA amplification | forward (EcoR I) |
| 1 | 5’-AATTAAGAATTCGCGCCCCGAACC-3’ |
| 2 | 5’-AAGTTAGAATTCCTGACCGAGACCTGG-3’ |
| 3 | 5’-AATATAGAATTCGGGGCCCTGGCCCTG -3’ |
| 4 | 5’-AGTCTAGAATTCGCCCTGACCAGACCTGG-3’ |
| 5 | 5’-ATATAAGAATTCGGGGCCCTGGCCCTGACC-3’ |
| 6 | 5’-AATATAGAATTCATGCAGCCGAGGTGGGCC-3’ |
| 7 | 5’-AATATAGAATTCGCCCTGACCGAGACCTGGGCC-3’ |
| 8 | 5’-AATATAGAATTCGCGCCCCGAACCCTCCTCCTG-3’ |
| 9 | 5’-ATTATAGAATTCCTGGCCCTGACCCAGACCTGG-3’ |
| 10 | 5’-AATATAGAATTCATGCAGCCGAGGTGGGCCCAA-3’ |
| 11 | 5’-AGCTTCGAATTCATGGCCCTGTCCTTTTCTTTA -3’ |
|  | reverse (Not I) |
| 12 | 5’-ACTAGAGCGGCCGCTTTTTTTTTTTTTTTTTTTTTTTTTTTTTT-3’ |
| cDNA library sequencing | Gp130 primer (forward) |
| 13 | 5’-GGCATGGAGGCTGCGACTG-3’ |
| cDNA library identification | 3’LTR Primer (reverse) |
| 14 | 5’-TCGTCGACCACTGTGCTGGC-3’ |
| YIPF2/pMG1 | forward (EcoR I) |
| 15 | ACTGCGAATTCATGGCATCGGCCGACGA |
|  | Reverse (XbaI) |
| 16 | TACGAGTCTAGATAGGAGGGGGCCAGGGAC |
| CD147EP/pSEL1 | forward (SaI I) |
| 17 | AAGTAGCGTCGACGGCTGCCGGCACAGTC |
|  | reverse (Not I) |
| 18 | TAATAGCGGCCGCTTAGTGGCTGCGCACGCG |
| CD147IP/pSEL1 | forward (SaI I) |
| 19 | ACATATGTCGACGAAGCGCCGGAAG |
|  | reverse (Not I) |
| 20 | TAATAGCGGCCGCGAGGTGAGAAC |

**
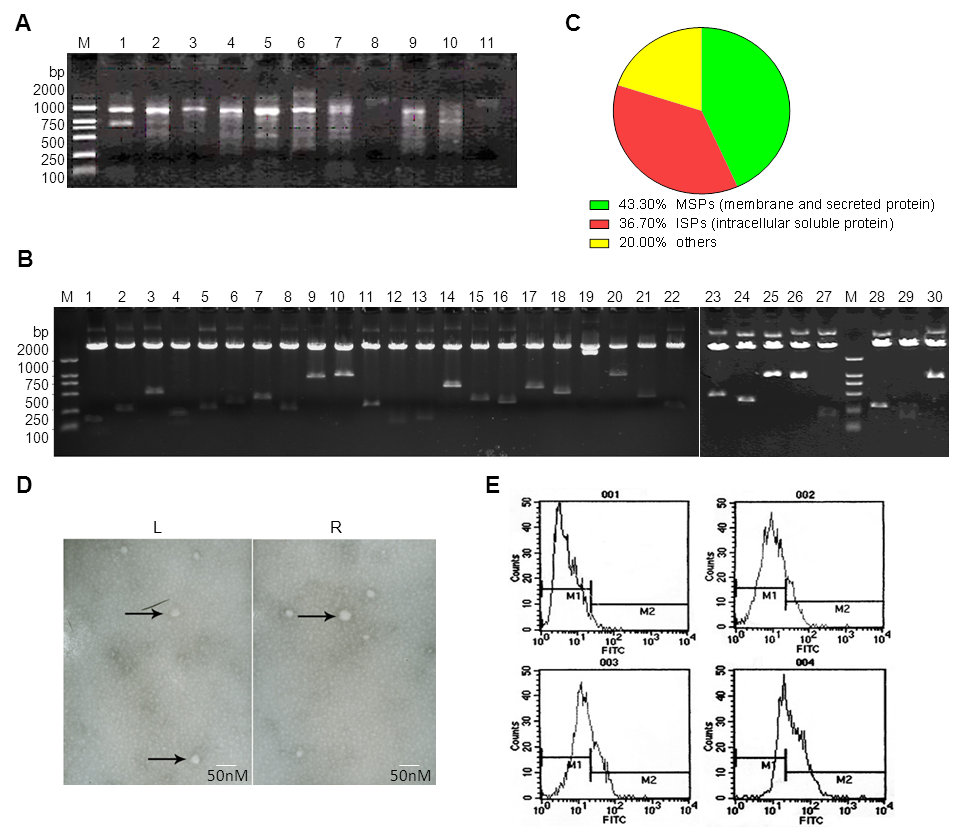
**

**Supplemental Fig. 1 Construction and identification of a retrovirus MSP-cDNA library**. **a,** Agarose electrophoresis analysis of amplified MSP-cDNA products. M: DL2000 DNA marker; 1-11: PCR products amplified by 11 pairs of primers listed in Tab S1. **b,** Agarose electrophoresis analysis of double-digested (NotI+EcoRI) plasmids extracted from the MSP-cDNA library. M: DL2000 DNA marker; 1-30: 30 random-selected clones; **c,** ORF constitutional analysis of the MSP-cDNA library. Sequencing results from randomly selected clones were submitted for BLAST analysis (http://blast.ncbi.nlm.nih.gov/Blast.cgi). **d,** Representative electron microscope imaging of retrovirus particles. L: the retroviral cDNA library packed by pBG1 plasmid; R: retroviral cDNA library packed by pBG1/MSP-cDNA plasmids. **e,** FACS analysis of viral titer of the packed retroviral MSP-cDNA library. HEK293-16 cells were infected with the serial diluted (001-004: 1/40, 1/4, 1/1, not diluted) retrovirus library mixed with pBG1/EGFP-packed virus, and the titer of the retrovirus library was estimated by EGFP expression level.


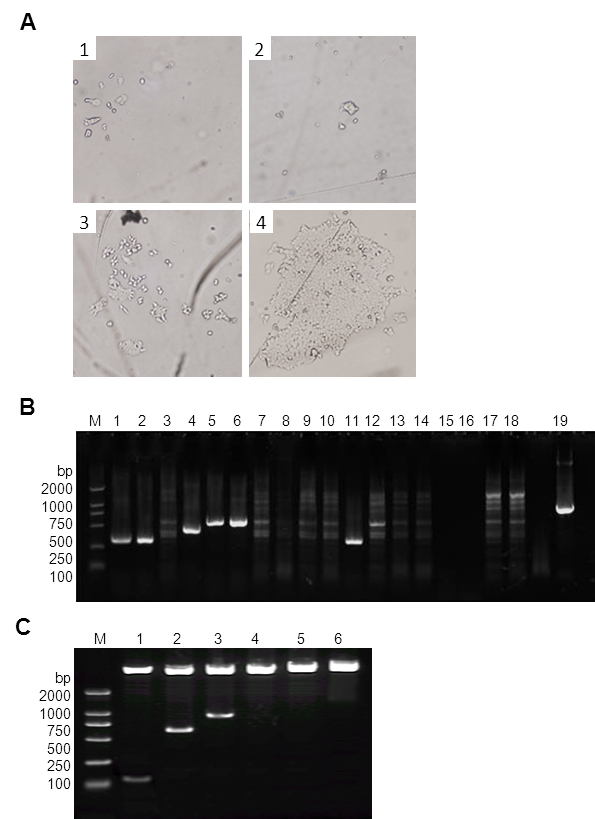


**Supplemental Fig. 2 Selection of positive clones after MAPPIT screening**. **a,** Reprehensive microscope imaging of cell clones that survived under puro (1ug/mL) and Epo (5ng/mL) selection. 1-4: treatment for 1-4 weeks, respectively. **b,** Agarose electrophoresis analysis of PCR-amplified products from screened clones. The candidate gene contained in surviving clones was amplified by PCR using primers as listed in Tab S1. M: DL2000 DNA marker; 1-18: 18 random-selected cell clones.19: recombinant pBG1-cccdB plasmid, positive control. **c,** Agarose electrophoresis analysis of recombinant plasmids with/without enzyme digestion. M: DL2000 DNA marker; 1, 4: pSEL1/CD147IP; 2, 5: pSEL1/CD147EP; 3, 6: pMG1/YIPF2; 1, 2: Sal1+Not1 double-digestion; 3: XhoI+XbaI double-digestion.


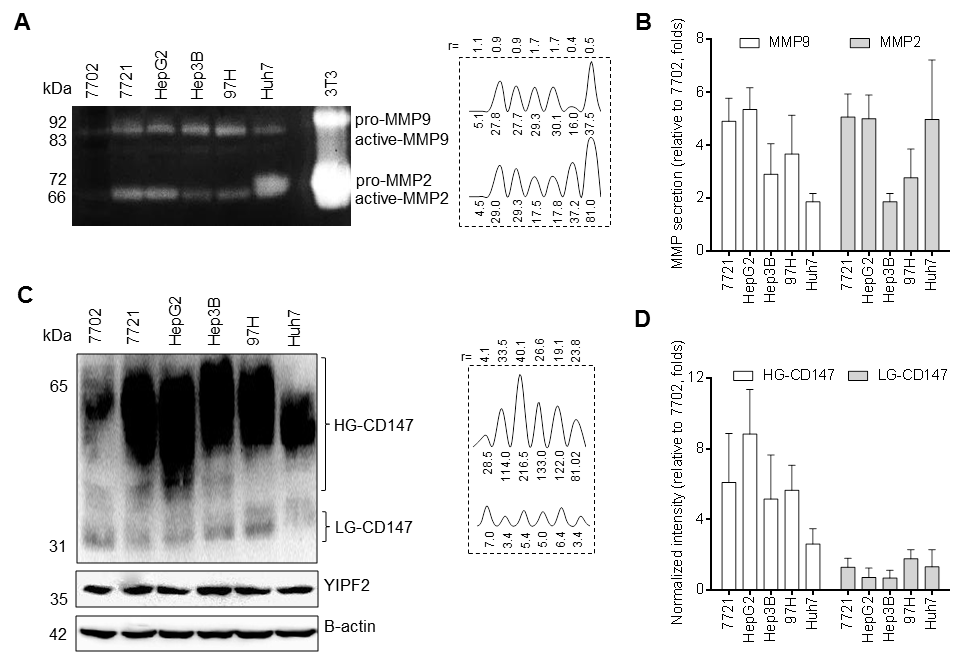


**Supplemental Fig. 3 Identifying the MMP secretion and CD147 expression in HCC cells**. **a,** Gelatin zymograph analysis of MMP2 and MMP9 secretion in HCC cells. The 3T3 cell was the positive control. The supernatants were concentrated by 40 fold using Amicon Ultra-4 10k device (Millipore). Equal volume protein samples were loaded. **c,** Western blot analysis of the expression level of endogenous CD147 and YIPF2 among indicated cell lines. Equal amounts of protein samples were loaded. Representative results from three independent experiments are shown (**a**, **c**), protein bands were quantified by Image J software, and corresponding quantitative data were analyzed (**b**, **d**).


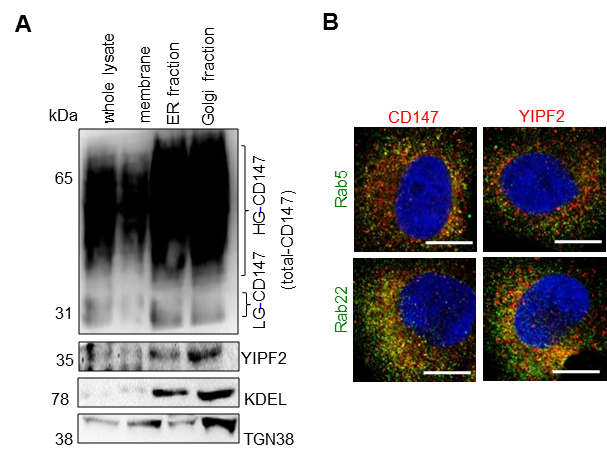


**Supplemental Fig. 4 Subcellular localization of YIPF2 and CD147**. **a,** Determining expression of YIPF2 and CD147 in the membrane, ER and Golgi fractions of HepG2 cells. Representative Western blot results from three independent experiments are shown. **b,** Confocal imaging the endosome localizations of YIPF2 and CD147. HepG2 cells were PFA-fixated, samponi-permeabilized, and stained with Ab combinations: anti-CD147 pcAb (red color, left column) or anti-YIPF2 Ab (red color, right column) together with anti-Rab5 Ab (green color, upper panel) and anti-Rab22 Ab (green color, lower panel), respectively. Representative observations are shown. Scale bar: 20 um.


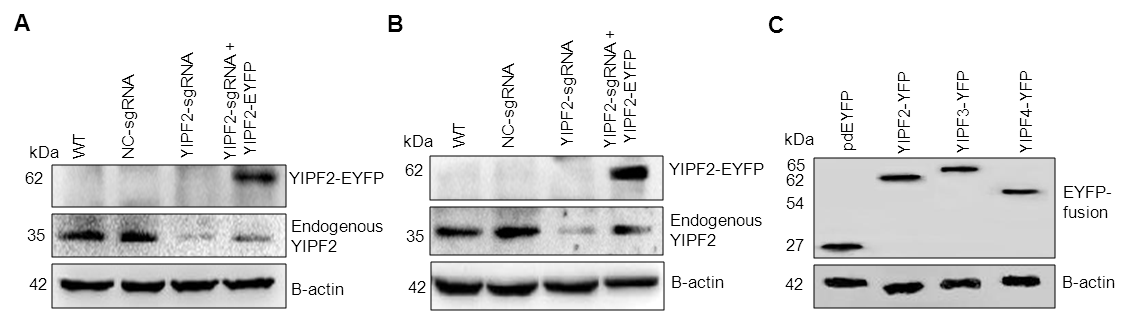


**Supplemental Fig. 5 Stable knock-down and transient overexpression of YIPF2**. HepG2 (**a**) and 7721 (**b**) cells were transfected with the YIPF2-sgRNA lentivirus, or further transfected with the YIPF2/pdEYFP plasmid. The NC-sgRNA lentivirus was used as a control. The stable knock-down and rescued expression of YIPF2 was determined by Western blotting. (**c**) HepG2 cells were transfected with YIPF2/YIPF3/YIPF4/pdEYFP plasmids, . Expression of EYFP-fused proteins was determined by Western blotting.


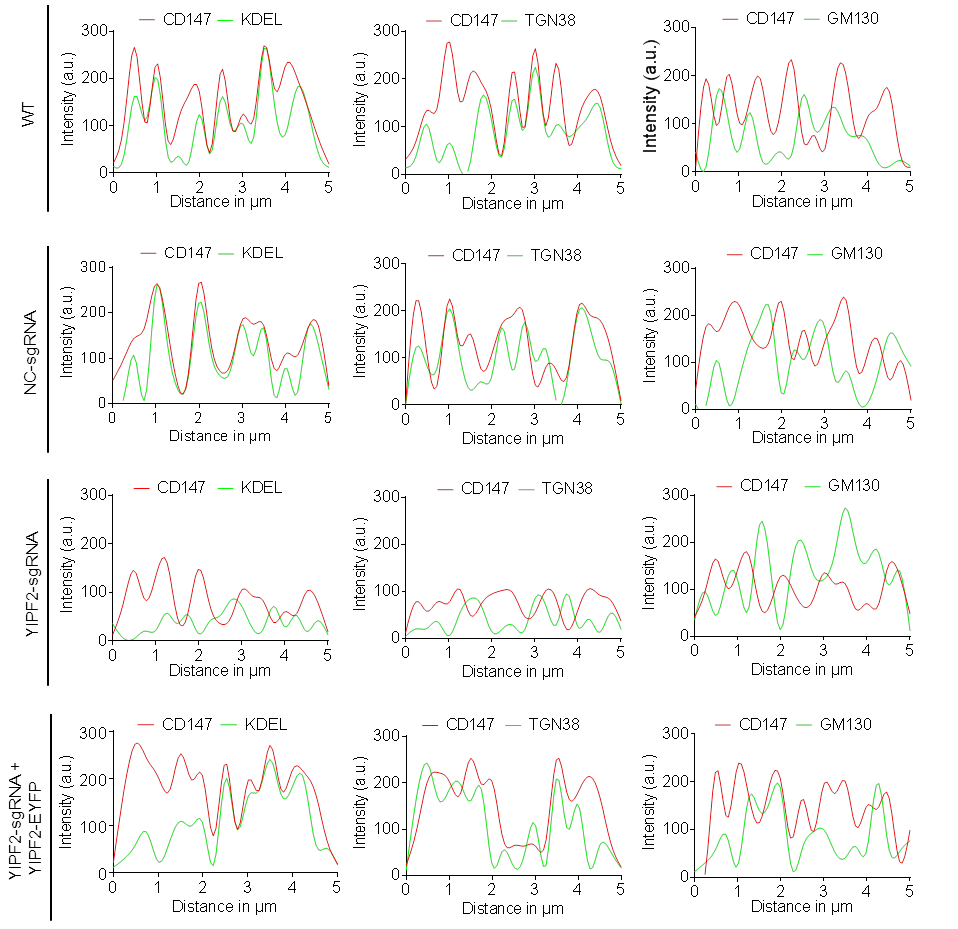


**Supplemental Fig. 6 Co-localization profiles of CD147 with ER/Golgi markers**. Line profiles show the arbitrary fluorescent intensity along the white lines in **Fig.4a**. a.u. denotes arbitrary unit. Legend: Red lines correspond to CD147 immunostaining, and green lines correspond to the immunostaining of KDEL, GM130, or TGN38.


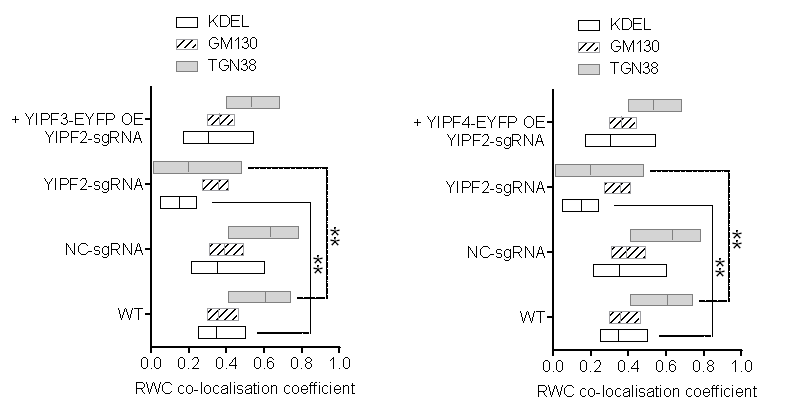


**Supplemental Fig. 7 RWC values of CD147 co-localized with ER/Golgi markers after YIPF3 (a) and YIPF4 (b) overexpression in YIPF2-KD HepG2 cells**.


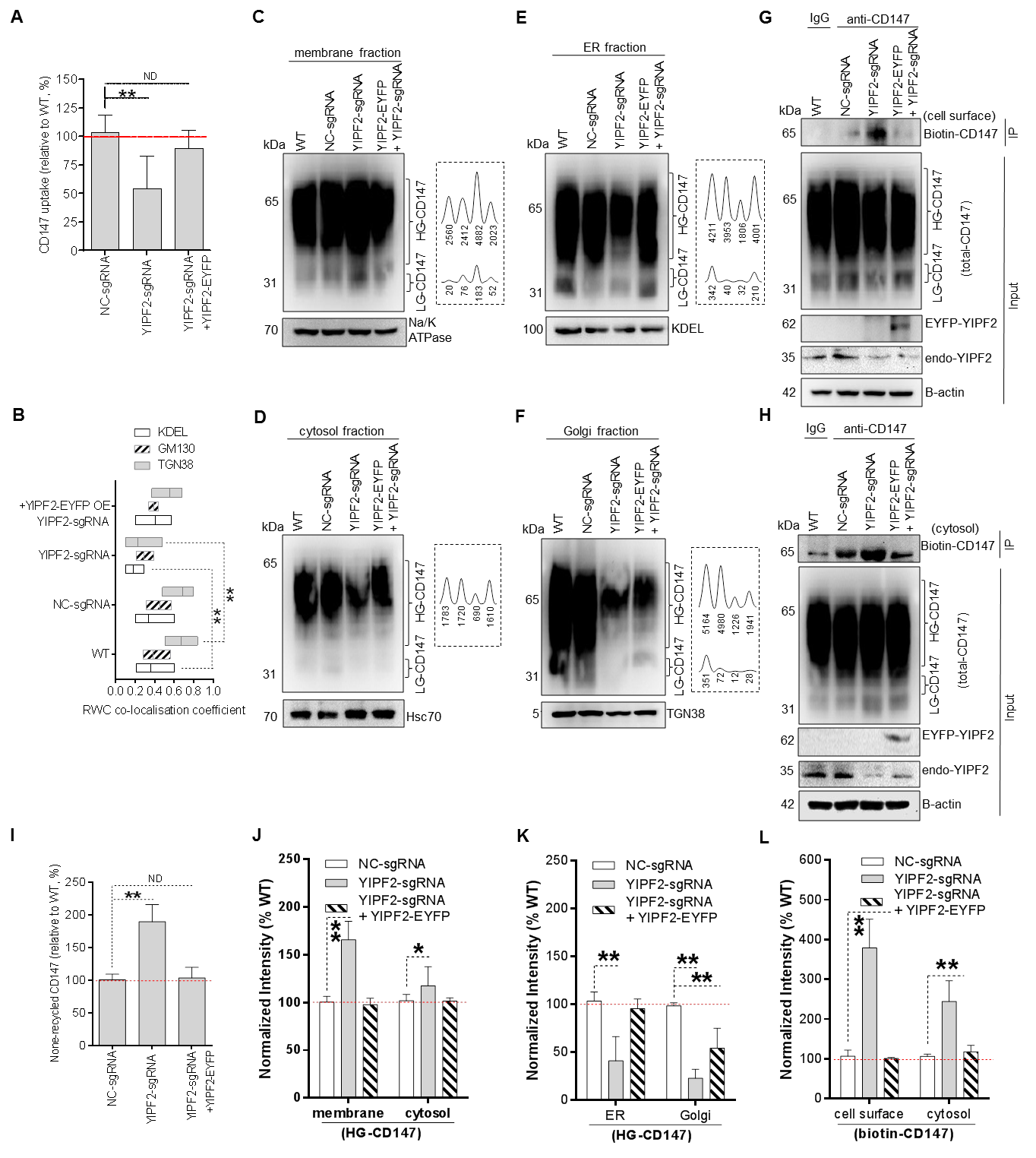


**Supplemental Fig. 8 YIPF2 regulates the endocytosis, ER-Golgi trafficking, glycosylation, and recycling of CD147**. YIPF2-KD 7721 cells (NC-KD cells as controls. WT: non-transfected 7721 cells) were transfected with the YIPF2/pdEYFP plasmid, then incubated with the H18Ab-AF488 complex at 37°C for uptake or recycling as previouslu described . **a,** The uptake of the H18Ab-AF488 complex in cells was quantified by flow cytometry. **b,** Confocal imaging of the co-localization of CD147 with the ER marker (KDEL) or Golgi markers (GM130, TGN38). Rank weighted coefficient (RWC) co-localization values of CD147 with KDEL, GM130, and TGN38 were quantified. **c**, **d**, **e**, **f,** Western blot determined the HG- and LG-CD147 from different fractions of transfected 7721 cells using H18 Ab. Quantitative scans of CD147 blots are presented at the right. **i,** The intracellular no-recycling portion of CD147 in 7721 cells was quantified by flow cytometry. Western blot determined the surface-resident biontin-CD147 pool (**g**) and intracellular non-recycled biotin-CD147 pool (**h**) after cell-surface biotinylation and anti-CD147 immunoprecipitation. Representative blot results from three independent experiments are shown (**c**-**h**), protein bands were quantified by Image J software, and corresponding quantitative data were analyzed (**j**, **k**, **l**). Statistically significant differences compared with NC-KD cells are shown: n=3, ** *P* <0.01.


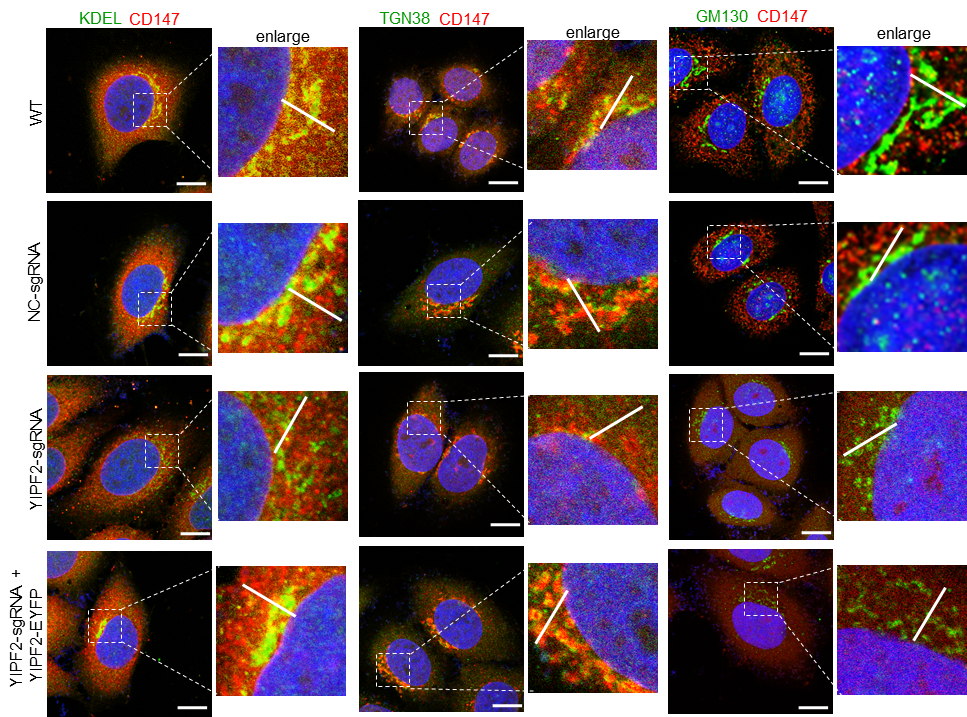


**Supplemental Fig. 9 YIPF2 knock-down dissipated ER-/Gogli-localized CD147.** YIPF2-KD 7721 cells (NC-KD cells as control. WT: non-transfected 7721 cells) were PFA-fixed, samponi-permeabilized, and stained by Ab combinations: anti-CD147 pcAb together with anti-KDEL, anti-GM130, and anti-TGN38 Abs, respectively (both plus corresponding anti-rabbit/mouse Ab-fluorescence ). Representative confocal observations are shown in the merged model. Scale bar: 20 um.


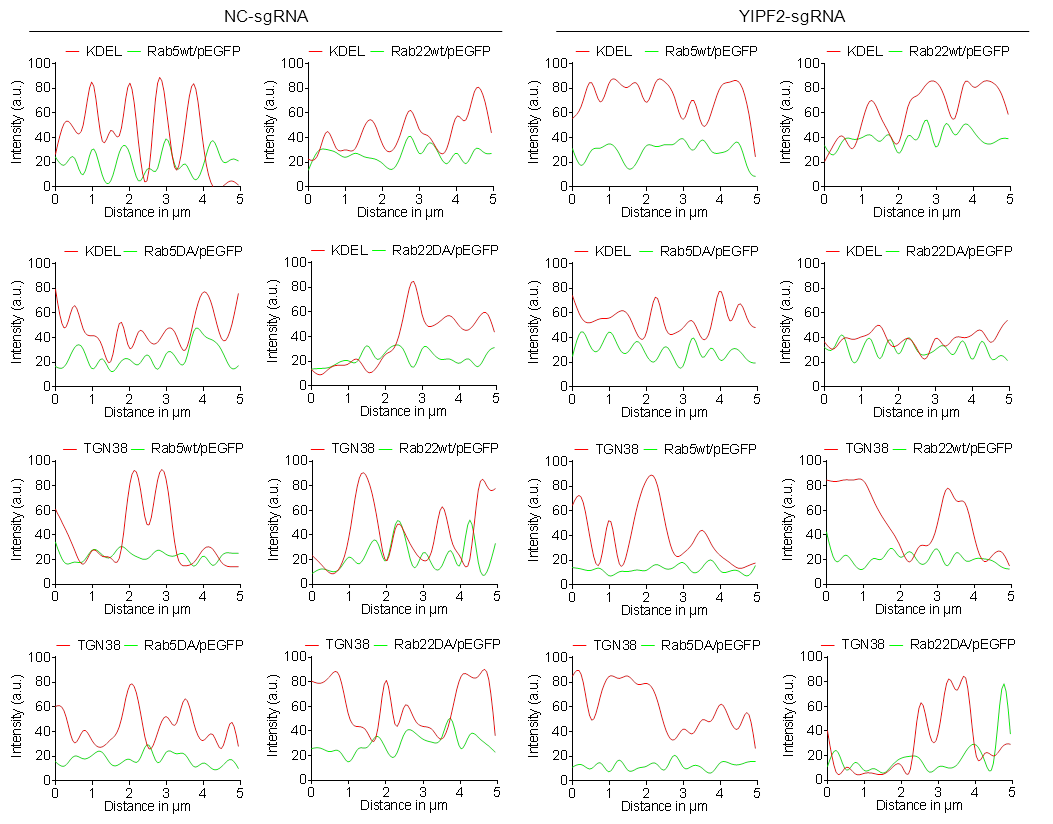


**Supplemental Fig. 10 Co-localization profiles of Rab5/Rab22a with ER/Golgi markers**. Line profiles show the arbitrary fluorescent intensity along the white lines in **Fig.6i**. a.u. denotes arbitrary unit. Legend: Red lines correspond to the immunostaining of KDEL/TGN38 (ER/Golgi markers), and green lines correspond to the immunostaining of wt-/DA-forms of Rab5/Rab22a.


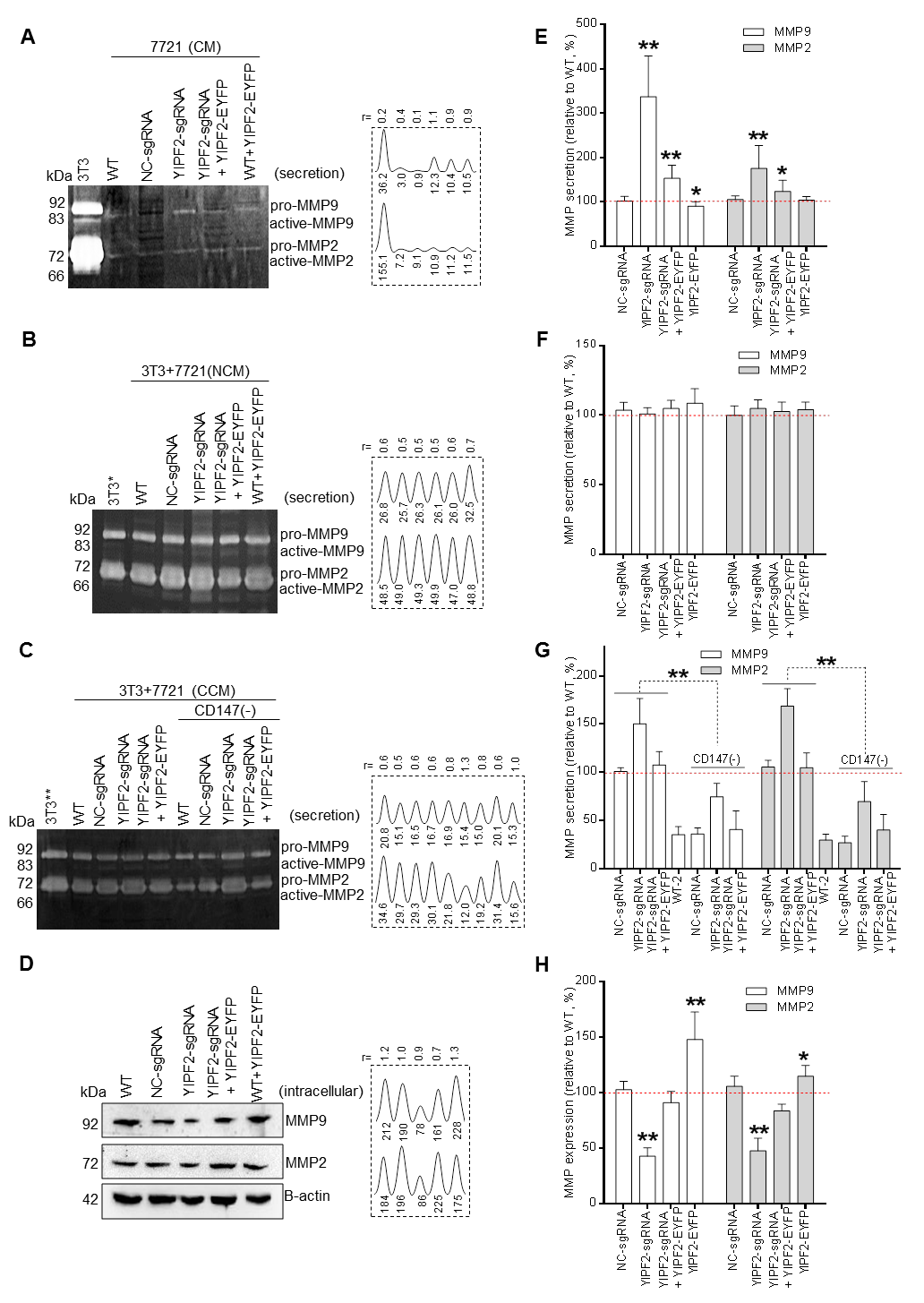


**Supplemental Fig. 11 YIPF2 knock-down increased MMP secretion in 7721 cells**. YIPF2-KD 7721 cells were transfected with the YIPF2/pEYFP plasmid for 48 hours culture. MMP activity (**a**-**c, e**-**g**) and endogenous MMP level (**d**, **h**) from different cultures were calculated as previously described . Representative results from three independent experiments are shown (**a**-**d**). Numbers below indicate corresponding areas of MMP peaks, and r values indicate MMP9/MMP2 ratios. MMP bands were quantified by Image J software, and corresponding quantitative data were analyzed (**e**-**h**). Statistically significant differences compared with NC-KD cells are shown: n=3, ** *P* <0.01, * *P* <0.05.
