# Supplementary material for: YIPF2 is a novel Rab-GDF that enhances HCC malignant phenotypes by facilitating CD147 endocytic recycle
Source: Cell Death Dis. 2019 Jun 12;10(6):462. doi: 10.1038/s41419-019-1709-8 (PMC6561952; doi:10.1038/s41419-019-1709-8)
Supplement: Supplementary file 2 — PCR primers used in this study [file 41419_2019_1709_MOESM2_ESM.docx]

**Table S1.** PCR primers used in this study.

| Primers | Sequence |
| --- | --- |
| MSPs-cDNA amplification | forward (EcoR I) |
| 1 | 5’-AATTAAGAATTCGCGCCCCGAACC-3’ |
| 2 | 5’-AAGTTAGAATTCCTGACCGAGACCTGG-3’ |
| 3 | 5’-AATATAGAATTCGGGGCCCTGGCCCTG -3’ |
| 4 | 5’-AGTCTAGAATTCGCCCTGACCAGACCTGG-3’ |
| 5 | 5’-ATATAAGAATTCGGGGCCCTGGCCCTGACC-3’ |
| 6 | 5’-AATATAGAATTCATGCAGCCGAGGTGGGCC-3’ |
| 7 | 5’-AATATAGAATTCGCCCTGACCGAGACCTGGGCC-3’ |
| 8 | 5’-AATATAGAATTCGCGCCCCGAACCCTCCTCCTG-3’ |
| 9 | 5’-ATTATAGAATTCCTGGCCCTGACCCAGACCTGG-3’ |
| 10 | 5’-AATATAGAATTCATGCAGCCGAGGTGGGCCCAA-3’ |
| 11 | 5’-AGCTTCGAATTCATGGCCCTGTCCTTTTCTTTA -3’ |
|  | reverse (Not I) |
| 12 | 5’-ACTAGAGCGGCCGCTTTTTTTTTTTTTTTTTTTTTTTTTTTTTT-3’ |
| cDNA library sequencing | Gp130 primer (forward) |
| 13 | 5’-GGCATGGAGGCTGCGACTG-3’ |
| cDNA library identification | 3’LTR Primer (reverse) |
| 14 | 5’-TCGTCGACCACTGTGCTGGC-3’ |
| YIPF2/pMG1 | forward (EcoR I) |
| 15 | ACTGCGAATTCATGGCATCGGCCGACGA |
|  | Reverse (XbaI) |
| 16 | TACGAGTCTAGATAGGAGGGGGCCAGGGAC |
| CD147EP/pSEL1 | forward (SaI I) |
| 17 | AAGTAGCGTCGACGGCTGCCGGCACAGTC |
|  | reverse (Not I) |
| 18 | TAATAGCGGCCGCTTAGTGGCTGCGCACGCG |
| CD147IP/pSEL1 | forward (SaI I) |
| 19 | ACATATGTCGACGAAGCGCCGGAAG |
|  | reverse (Not I) |
| 20 | TAATAGCGGCCGCGAGGTGAGAAC |
