# Supplementary material for: YIPF2 is a novel Rab-GDF that enhances HCC malignant phenotypes by facilitating CD147 endocytic recycle
Source: Cell Death Dis. 2019 Jun 12;10(6):462. doi: 10.1038/s41419-019-1709-8 (PMC6561952; doi:10.1038/s41419-019-1709-8)
Supplement: Supplementary file 3 — Construction and identification of a retrovirus MSP-cDNA library [file 41419_2019_1709_MOESM3_ESM.docx]

**
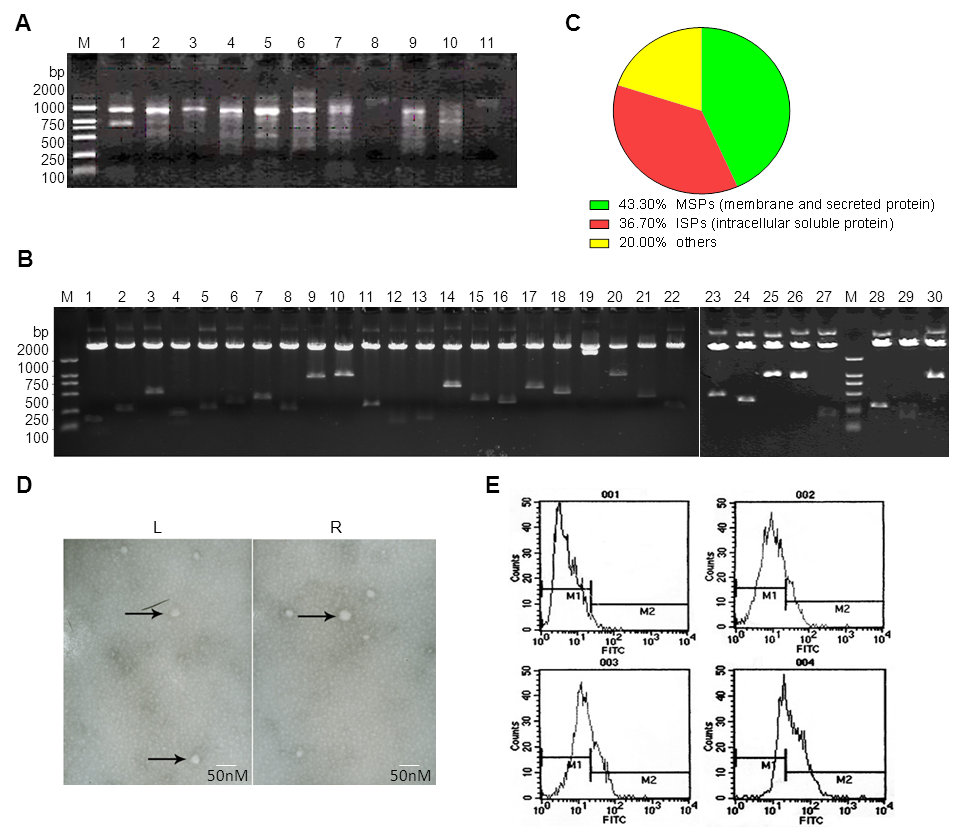
**

**Supplemental Fig. 1 Construction and identification of a retrovirus MSP-cDNA library**. **a**, Agarose electrophoresis analysis of amplified MSP-cDNA products. M: DL2000 DNA marker; 1-11: PCR products amplified by 11 pairs of primers listed in Tab S1. **b**, Agarose electrophoresis analysis of double-digested (NotI+EcoRI) plasmids extracted from the MSP-cDNA library. M: DL2000 DNA marker; 1-30: 30 random-selected clones; **c,** ORF constitutional analysis of the MSP-cDNA library. Sequencing results from randomly selected clones were submitted for BLAST analysis (http://blast.ncbi.nlm.nih.gov/Blast.cgi). **d**, Representative electron microscope imaging of retrovirus particles. L: the retroviral cDNA library packed by pBG1 plasmid; R: retroviral cDNA library packed by pBG1/MSP-cDNA plasmids. **e,** FACS analysis of viral titer of the packed retroviral MSP-cDNA library. HEK293-16 cells were infected with the serial diluted (001-004: 1/40, 1/4, 1/1, not diluted) retrovirus library mixed with pBG1/EGFP-packed virus, and the titer of the retrovirus library was estimated by EGFP expression level.
