# Supplementary material for: YIPF2 is a novel Rab-GDF that enhances HCC malignant phenotypes by facilitating CD147 endocytic recycle
Source: Cell Death Dis. 2019 Jun 12;10(6):462. doi: 10.1038/s41419-019-1709-8 (PMC6561952; doi:10.1038/s41419-019-1709-8)
Supplement: Supplementary file 4 — Selection of positive clones after MAPPIT screening [file 41419_2019_1709_MOESM4_ESM.docx]

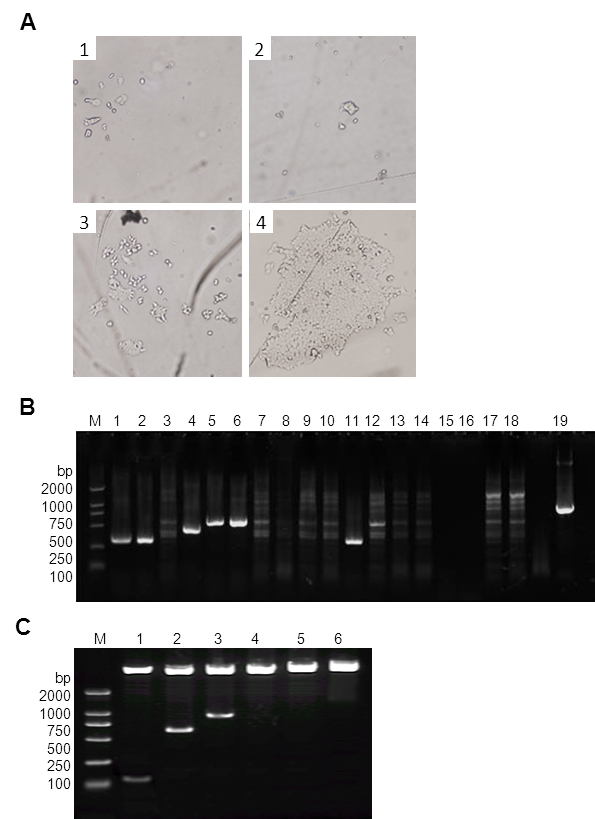


**Supplemental Fig. 2 Selection of positive clones after MAPPIT screening**. **a**, Reprehensive microscope imaging of cell clones that survived under puro (1ug/mL) and Epo (5ng/mL) selection. 1-4: treatment for 1-4 weeks, respectively. **b**, Agarose electrophoresis analysis of PCR-amplified products from screened clones. The candidate gene contained in surviving clones was amplified by PCR using primers as listed in Tab S1. M: DL2000 DNA marker; 1-18: 18 random-selected cell clones.19: recombinant pBG1-cccdB plasmid, positive control. **c**, Agarose electrophoresis analysis of recombinant plasmids with/without enzyme digestion. M: DL2000 DNA marker; 1, 4: pSEL1/CD147IP; 2, 5: pSEL1/CD147EP; 3, 6: pMG1/YIPF2; 1, 2: Sal1+Not1 double-digestion; 3: XhoI+XbaI double-digestion.
