# Supplementary material for: YIPF2 is a novel Rab-GDF that enhances HCC malignant phenotypes by facilitating CD147 endocytic recycle
Source: Cell Death Dis. 2019 Jun 12;10(6):462. doi: 10.1038/s41419-019-1709-8 (PMC6561952; doi:10.1038/s41419-019-1709-8)
Supplement: Supplementary file 5 — Identifying the MMP secretion and CD147 expression in HCC cells [file 41419_2019_1709_MOESM5_ESM.docx]

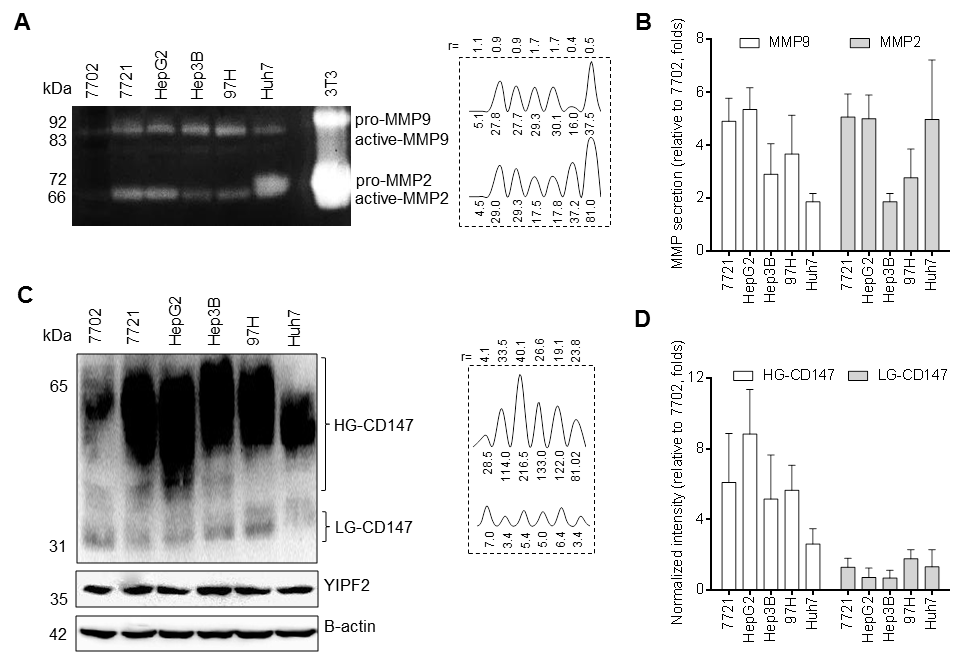


**Supplemental Fig. 3 Identifying the MMP secretion and CD147 expression in HCC cells**. **a**, Gelatin zymograph analysis of MMP2 and MMP9 secretion in HCC cells. The 3T3 cell was the positive control. The supernatants were concentrated by 40 fold using Amicon Ultra-4 10k device (Millipore). Equal volume protein samples were loaded. **c,** Western blot analysis of the expression level of endogenous CD147 and YIPF2 among indicated cell lines. Equal amounts of protein samples were loaded. Representative results from three independent experiments are shown (**a**, **c**), protein bands were quantified by Image J software, and corresponding quantitative data were analyzed (**b**, **d**).
