# Supplementary material for: YIPF2 is a novel Rab-GDF that enhances HCC malignant phenotypes by facilitating CD147 endocytic recycle
Source: Cell Death Dis. 2019 Jun 12;10(6):462. doi: 10.1038/s41419-019-1709-8 (PMC6561952; doi:10.1038/s41419-019-1709-8)
Supplement: Supplementary file 6 — Subcellular localization of YIPF2 and CD147 [file 41419_2019_1709_MOESM6_ESM.docx]

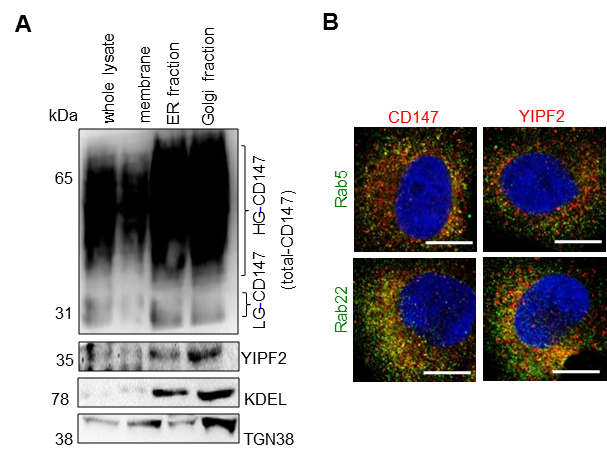


**Supplemental Fig. 4 Subcellular localization of YIPF2 and CD147**. **a,** Determining expression of YIPF2 and CD147 in the membrane, ER and Golgi fractions of HepG2 cells. Representative Western blot results from three independent experiments are shown. **b**, Confocal imaging the endosome localizations of YIPF2 and CD147. HepG2 cells were PFA-fixated, samponi-permeabilized, and stained with Ab combinations: anti-CD147 pcAb (red color, left column) or anti-YIPF2 Ab (red color, right column) together with anti-Rab5 Ab (green color, upper panel) and anti-Rab22 Ab (green color, lower panel), respectively. Representative observations are shown. Scale bar: 20 um.
