# Supplementary material for: YIPF2 is a novel Rab-GDF that enhances HCC malignant phenotypes by facilitating CD147 endocytic recycle
Source: Cell Death Dis. 2019 Jun 12;10(6):462. doi: 10.1038/s41419-019-1709-8 (PMC6561952; doi:10.1038/s41419-019-1709-8)
Supplement: Supplementary file 7 — Stable knock-down and transient overexpression of YIPF2 [file 41419_2019_1709_MOESM7_ESM.docx]

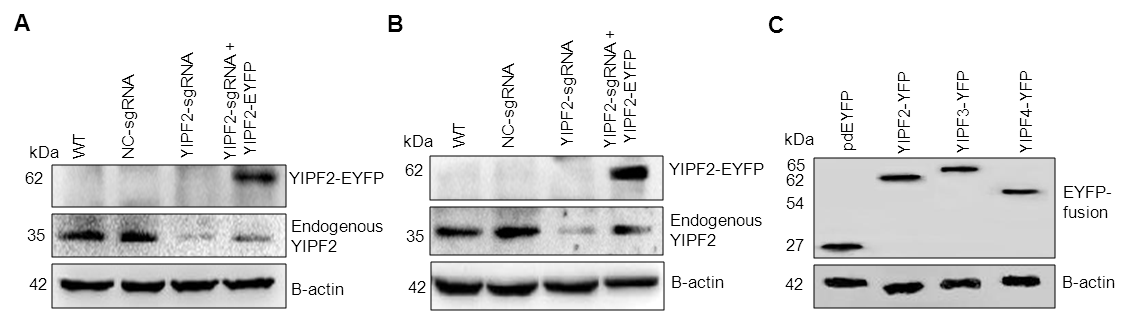


**Supplemental Fig. 5 Stable knock-down and transient overexpression of YIPF2**. HepG2 (**a**) and 7721 (**b**) cells were transfected with the YIPF2-sgRNA lentivirus, or further transfected with the YIPF2/pdEYFP plasmid. The NC-sgRNA lentivirus was used as a control. The stable knock-down and rescued expression of YIPF2 was determined by Western blotting. (**c**) HepG2 cells were transfected with YIPF2/YIPF3/YIPF4/pdEYFP plasmids. Expression of EYFP-fused proteins was determined by Western blotting.
