# Supplementary material for: YIPF2 is a novel Rab-GDF that enhances HCC malignant phenotypes by facilitating CD147 endocytic recycle
Source: Cell Death Dis. 2019 Jun 12;10(6):462. doi: 10.1038/s41419-019-1709-8 (PMC6561952; doi:10.1038/s41419-019-1709-8)
Supplement: Supplementary file 9 — RWC values of CD147 co-localized with ER/Golgi markers after YIPF3 (a) and YIPF4 (b) overexpression in YIPF2-KD HepG2 cells [file 41419_2019_1709_MOESM9_ESM.docx]

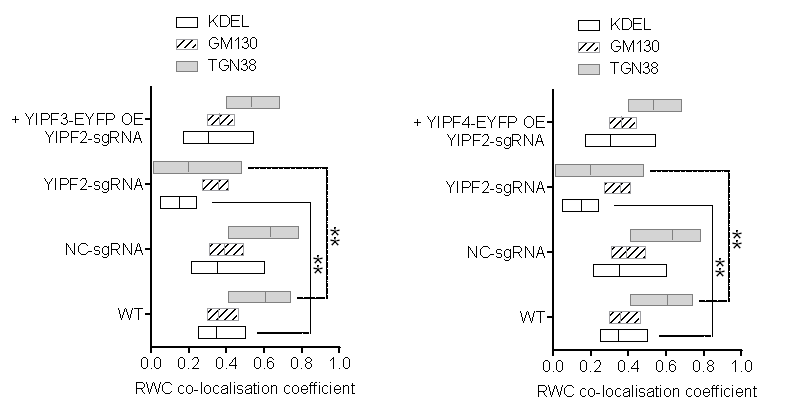


**Supplemental Fig. 7 RWC values of CD147 co-localized with ER/Golgi markers after YIPF3 (a) and YIPF4 (b) overexpression in YIPF2-KD HepG2 cells**.
