# Supplementary material for: YIPF2 is a novel Rab-GDF that enhances HCC malignant phenotypes by facilitating CD147 endocytic recycle
Source: Cell Death Dis. 2019 Jun 12;10(6):462. doi: 10.1038/s41419-019-1709-8 (PMC6561952; doi:10.1038/s41419-019-1709-8)
Supplement: Supplementary file 10 — YIPF2 regulates the endocytosis, ER-Golgi trafficking, glycosylation, and recycling of CD147 [file 41419_2019_1709_MOESM10_ESM.docx]

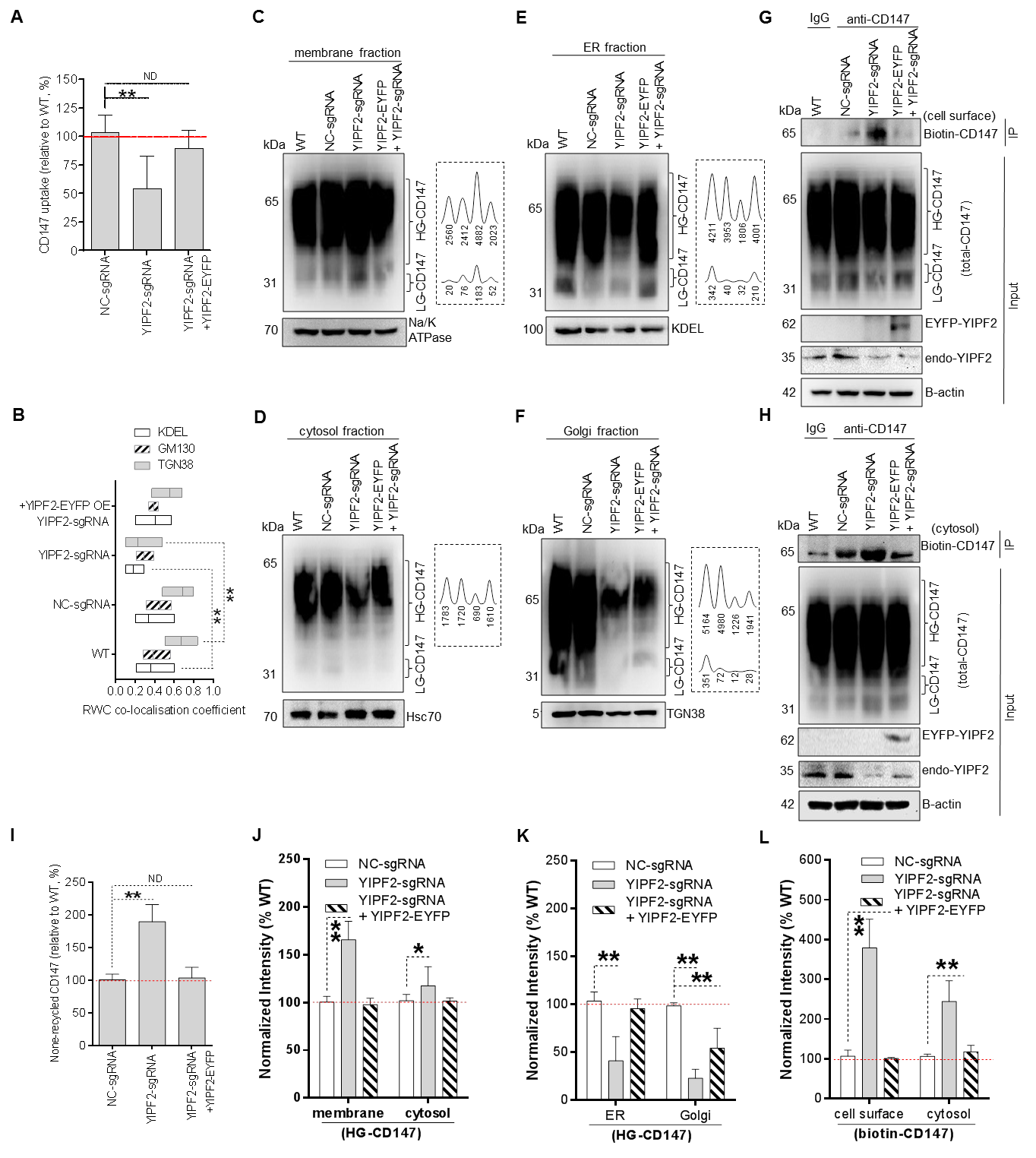


**Supplemental Fig. 8 YIPF2 regulates the endocytosis, ER-Golgi trafficking, glycosylation, and recycling of CD147**. YIPF2-KD 7721 cells (NC-KD cells as controls. WT: non-transfected 7721 cells) were transfected with the YIPF2/pdEYFP plasmid, then incubated with the H18Ab-AF488 complex at 37°C for uptake or recycling as previouslu described. **a**, The uptake of the H18Ab-AF488 complex in cells was quantified by flow cytometry. **b,** Confocal imaging of the co-localization of CD147 with the ER marker (KDEL) or Golgi markers (GM130, TGN38). Rank weighted coefficient (RWC) co-localization values of CD147 with KDEL, GM130, and TGN38 were quantified. **c**, **d**, **e**, **f**, Western blot determined the HG- and LG-CD147 from different fractions of transfected 7721 cells using H18 Ab. Quantitative scans of CD147 blots are presented at the right. **i**, The intracellular no-recycling portion of CD147 in 7721 cells was quantified by flow cytometry. Western blot determined the surface-resident biontin-CD147 pool (**g**) and intracellular non-recycled biotin-CD147 pool (**h**) after cell-surface biotinylation and anti-CD147 immunoprecipitation. Representative blot results from three independent experiments are shown (**c**-**h**), protein bands were quantified by Image J software, and corresponding quantitative data were analyzed (**j**, **k**, **l**). Statistically significant differences compared with NC-KD cells are shown: n=3, ** *P* <0.01.
