# Supplementary material for: YIPF2 is a novel Rab-GDF that enhances HCC malignant phenotypes by facilitating CD147 endocytic recycle
Source: Cell Death Dis. 2019 Jun 12;10(6):462. doi: 10.1038/s41419-019-1709-8 (PMC6561952; doi:10.1038/s41419-019-1709-8)
Supplement: Supplementary file 11 — YIPF2 knock-down dissipated ER-/Gogli-localized CD147 [file 41419_2019_1709_MOESM11_ESM.docx]

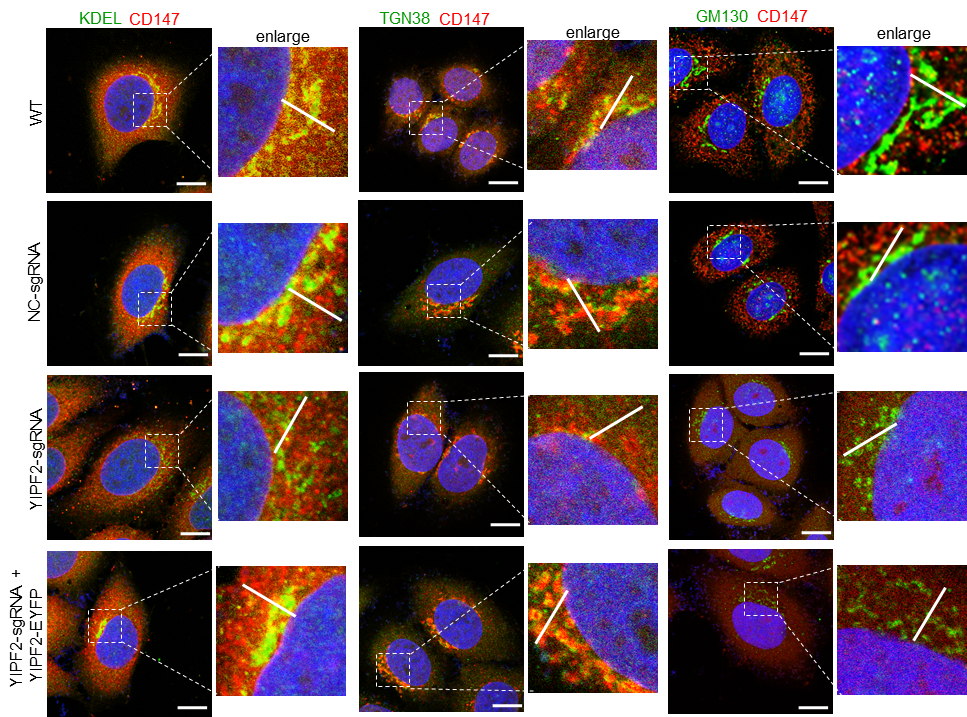


**Supplemental Fig. 9 YIPF2 knock-down dissipated ER-/Gogli-localized CD147.** YIPF2-KD 7721 cells (NC-KD cells as control. WT: non-transfected 7721 cells) were PFA-fixed, samponi-permeabilized, and stained by Ab combinations: anti-CD147 pcAb together with anti-KDEL, anti-GM130, and anti-TGN38 Abs, respectively (both plus corresponding anti-rabbit/mouse Ab-fluorescence). Representative confocal observations are shown in the merged model. Scale bar: 20 um.
