# Supplementary material for: YIPF2 is a novel Rab-GDF that enhances HCC malignant phenotypes by facilitating CD147 endocytic recycle
Source: Cell Death Dis. 2019 Jun 12;10(6):462. doi: 10.1038/s41419-019-1709-8 (PMC6561952; doi:10.1038/s41419-019-1709-8)
Supplement: Supplementary file 12 — Co-localization profiles of Rab5/Rab22a with ER/Golgi markers [file 41419_2019_1709_MOESM12_ESM.docx]

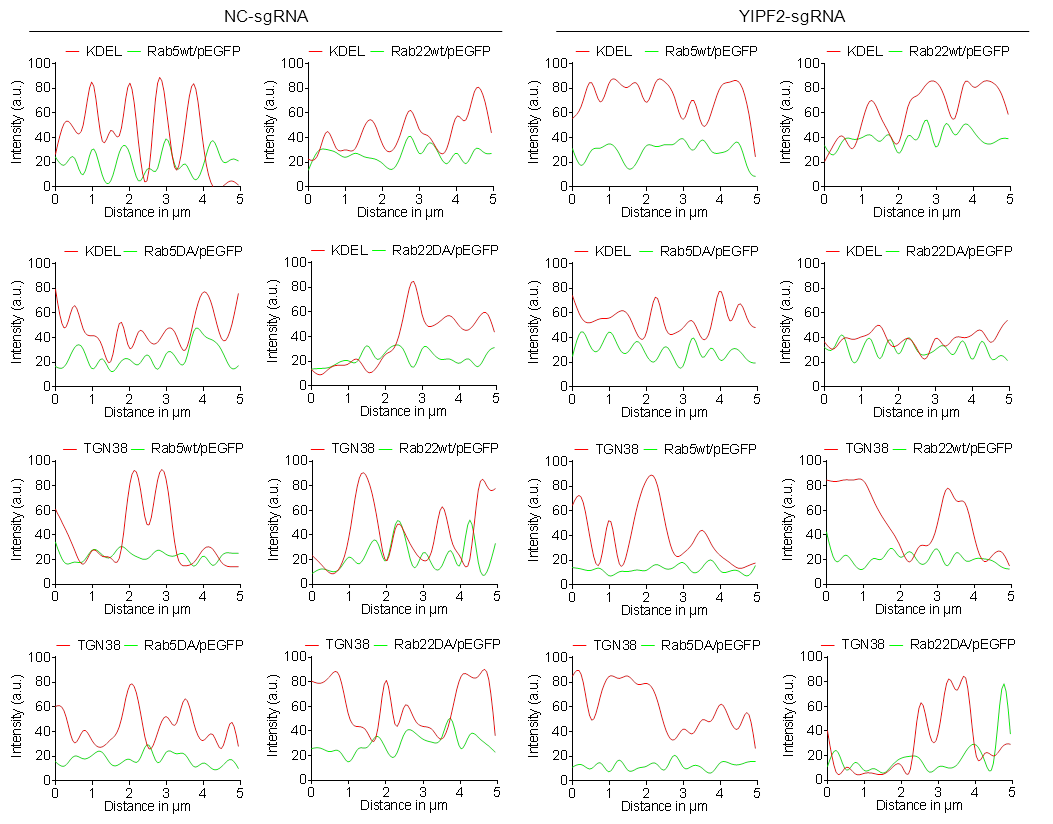


**Supplemental Fig. 10 Co-localization profiles of Rab5/Rab22a with ER/Golgi markers**. Line profiles show the arbitrary fluorescent intensity along the white lines in **Fig.6i**. a.u. denotes arbitrary unit. Legend: Red lines correspond to the immunostaining of KDEL/TGN38 (ER/Golgi markers), and green lines correspond to the immunostaining of wt-/DA-forms of Rab5/Rab22a.
