# Supplementary material for: YIPF2 is a novel Rab-GDF that enhances HCC malignant phenotypes by facilitating CD147 endocytic recycle
Source: Cell Death Dis. 2019 Jun 12;10(6):462. doi: 10.1038/s41419-019-1709-8 (PMC6561952; doi:10.1038/s41419-019-1709-8)
Supplement: Supplementary file 13 — YIPF2 knock-down increased MMP secretion in 7721 cells [file 41419_2019_1709_MOESM13_ESM.docx]

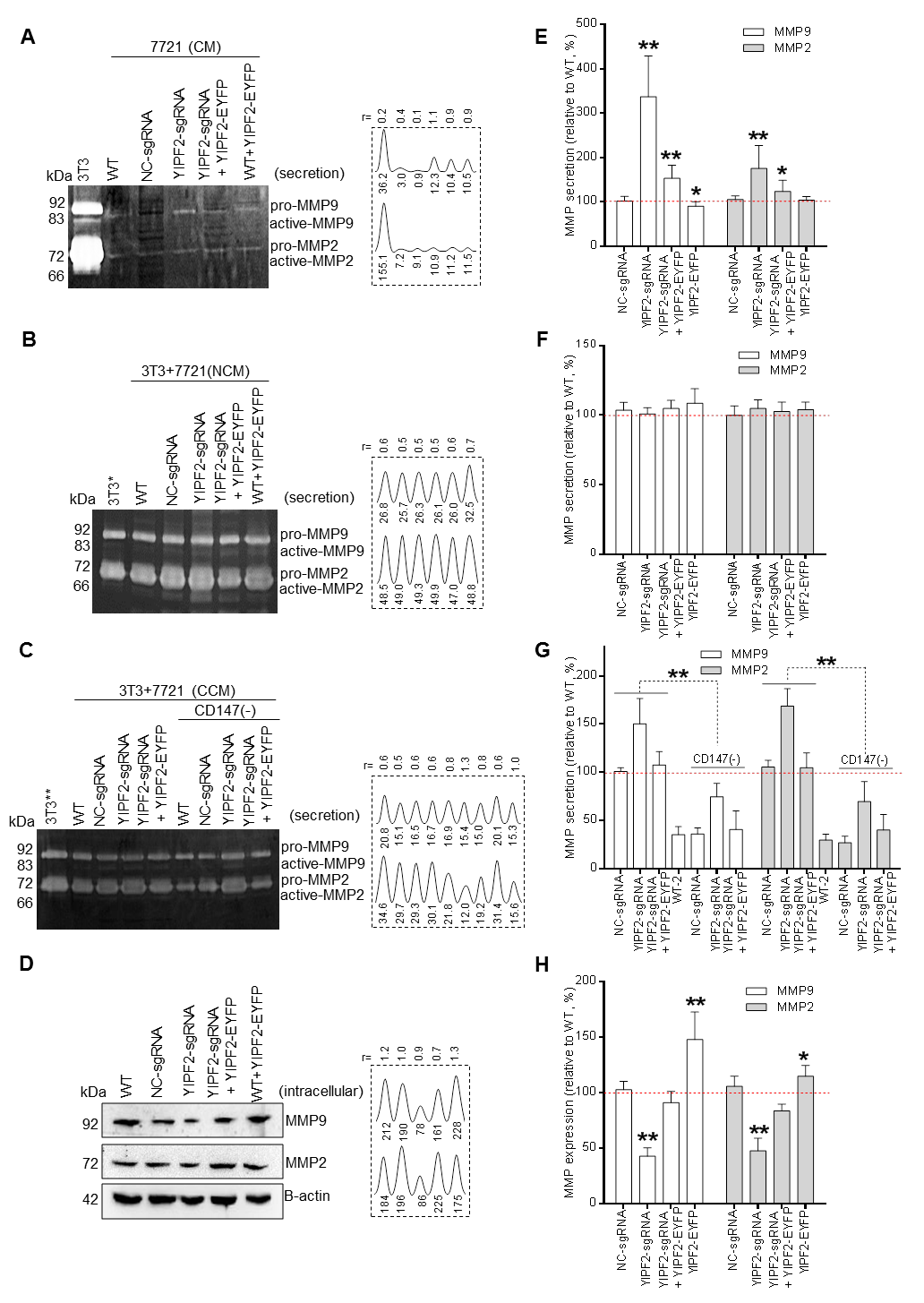


**Supplemental Fig. 11 YIPF2 knock-down increased MMP secretion in 7721 cells**. YIPF2-KD 7721 cells were transfected with the YIPF2/pEYFP plasmid for 48 hours culture. MMP activity (**a**-**c**, **e**-**g**) and endogenous MMP level (**d**, **h**) from different cultures were calculated as previously described. Representative results from three independent experiments are shown (**a**-**d**). Numbers below indicate corresponding areas of MMP peaks, and r values indicate MMP9/MMP2 ratios. MMP bands were quantified by Image J software, and corresponding quantitative data were analyzed (**e**-**h**). Statistically significant differences compared with NC-KD cells are shown: n=3, ** *P* <0.01, * *P* <0.05.
